# Dynamics of coral-associated bacterial communities acclimated to temperature stress based on recent thermal history

**DOI:** 10.1038/s41598-017-14927-3

**Published:** 2017-11-02

**Authors:** Jia-Ho Shiu, Shashank Keshavmurthy, Pei-Wen Chiang, Hsing-Ju Chen, Shueh-Ping Lou, Ching-Hung Tseng, Hernyi Justin Hsieh, Chaolun Allen Chen, Sen-Lin Tang

**Affiliations:** 1 Molecular and Biological Agricultural Sciences Program, Taiwan International Graduate Program, Academia Sinica, Taipei, Taiwan, and National Chung-Hsing University, Taichung, Taiwan; 2 Biodiversity Research Center, Academia Sinica, Taipei, Taiwan; 3 Graduate Institute of Biotechnology, National Chung-Hsing University, Taichung, Taiwan; 4 Germark Biotechnology Co., Ltd., Taichung, 40767 Taiwan; 5 Penghu Marine Biology Research Center, Fishery Research Institute, Council of Agriculture, Magong, Penghu, 880 Taiwan; 6 Biotechnology Center, National Chung-Hsing University, Taichung, Taiwan

## Abstract

Seasonal variation in temperature fluctuations may provide corals and their algal symbionts varying abilities to acclimate to changing temperatures. We hypothesized that different temperature ranges between seasons may promote temperature-tolerance of corals, which would increase stability of a bacterial community following thermal stress. *Acropora muricata* coral colonies were collected in summer and winter (water temperatures were 23.4–30.2 and 12.1–23.1 °C, respectively) from the Penghu Archipelago in Taiwan, then exposed to 6 temperature treatments (10–33 °C). Changes in coral-associated bacteria were determined after 12, 24, and 48 h. Based on 16S rRNA gene amplicons and Illumina sequencing, bacterial communities differed between seasons and treatments altered the dominant bacteria. Cold stress caused slower shifts in the bacterial community in winter than in summer, whereas a more rapid shift occurred under heat stress in both seasons. Results supported our hypothesis that bacterial community composition of corals in winter are more stable in cold temperatures but changed rapidly in hot temperatures, with opposite results for the bacterial communities in summer. We infer that the thermal tolerance ranges of coral-associated bacteria, with a stable community composition, are associated with their short-term (3 mo) seawater thermal history. Therefore, seasonal acclimation may increase tolerance of coral-associated bacteria to temperature fluctuations.

## Introduction

Coral-associated bacteria are critical to the health and function of coral ecosystems and coral holobiont^1,2^. Coral-associated bacteria are dynamic and often form stable homeostatic associations with their host^3^. Therefore, changes in these bacterial communities may be an indicator of health of coral holobionts^4^.

Increased temperature is a stressor for coral reefs and may adversely affect coral holobiont physiology^5,6^. Temperature stress from global climate change, often a critical factor in massive coral bleaching^7,8^, can directly or indirectly influence coral health, increasing susceptibility to disease or facilitating pathogen propagation^9,10^. Shifts in coral bacterial communities are common after heat stress^9,11–13^. Further to this, cold stress may also induce coral bleaching^14–16^ and cause mortality^17,18^. Cold stress may decrease growth, metabolic activity, respiration and chlorophyll *a* content in corals^16,19,20^. However, effects of cold stress on coral-associated bacterial communities have not been well characterized.

To reduce detrimental impacts of thermal stress corals may acclimatize^21,22^. Mechanisms responsible for physiological acclimation are not well understood, but the process is likely affected by thermal history^23,24^. Corals can resist temperature stress or acclimate to dramatic temperature fluctuations^25–28^. Interestingly, coral-associated bacterial communities also exhibit a similar phenomenon. After acclimatization, the bacterial community in corals may remain stable before and after heat stress^29,30^. Santos and co-workers (2014)^29^ preheated coral colonies prior to heat treatment; in the study, communities in heat-acclimatized samples were more stable than in those without pretreatment. Similarly, Ziegler and co-workers (2017)^30^ transplanted coral colonies to pools with higher temperature for acclimation before a heat-treatment experiment. After 1 y of acclimation, transplanted colonies had consistent bacterial communities before and after heat treatment, whereas colonies that had remained in original habitats had altered composition of bacterial communities after treatment.

Responses of coral-associated bacteria to acclimation, more specifically variation in responses to acute stress, have not been well characterized. Seasonal acclimation in coral-associated bacteria is unknown, as only between-season variations in the coral-associated bacterial community have been reported^31–35^. To better understand this phenomenon, we conducted a comprehensive study to determine effects of temperature stress on changes in bacteria with different seasonal thermal histories.

The objectives were to detect short-term dynamics in coral-associated bacterial communities under various temperatures and to compare this variation between summer and winter. *Acropora muricata* coral colonies were collected from the Penghu Archipelago, located in western Taiwan, proposed as a climate-change refuge for corals in a previous study^36^. It has a unique thermal regime; warm waters of the Kuroshio Current pass through the islands from the south in summer, whereas in winter, cold waters of the China Coastal Current move down the islands from the north. Therefore, this area has a wide range (18 °C) in temperatures annually (from 12.1 to 30.1 °C). Consequently, average monthly seawater temperature in Penghu ranges from 18.1 °C in winter to 27.7 °C in summer. Therefore, we conducted tank experiments with temperature treatments that compassing a range from 10 to 33 °C (2 °C lower than the lowest temperature in Penghu and 3 °C higher than the highest temperature), which were 10 °C, 15 °C, 20 °C, 25 °C /26 °C, 30 °C and 33 °C. This study is the first to characterize the impacts of cold and heat stressors on changes in coral-associated bacterial communities after seasonal acclimation (summer and winter). We concluded that seasonal thermal history may enhance stability of the bacterial community against thermal stress.

## Results

### Composition of bacterial communities between coral and seawater in 2 seasons

**Figure 1:**
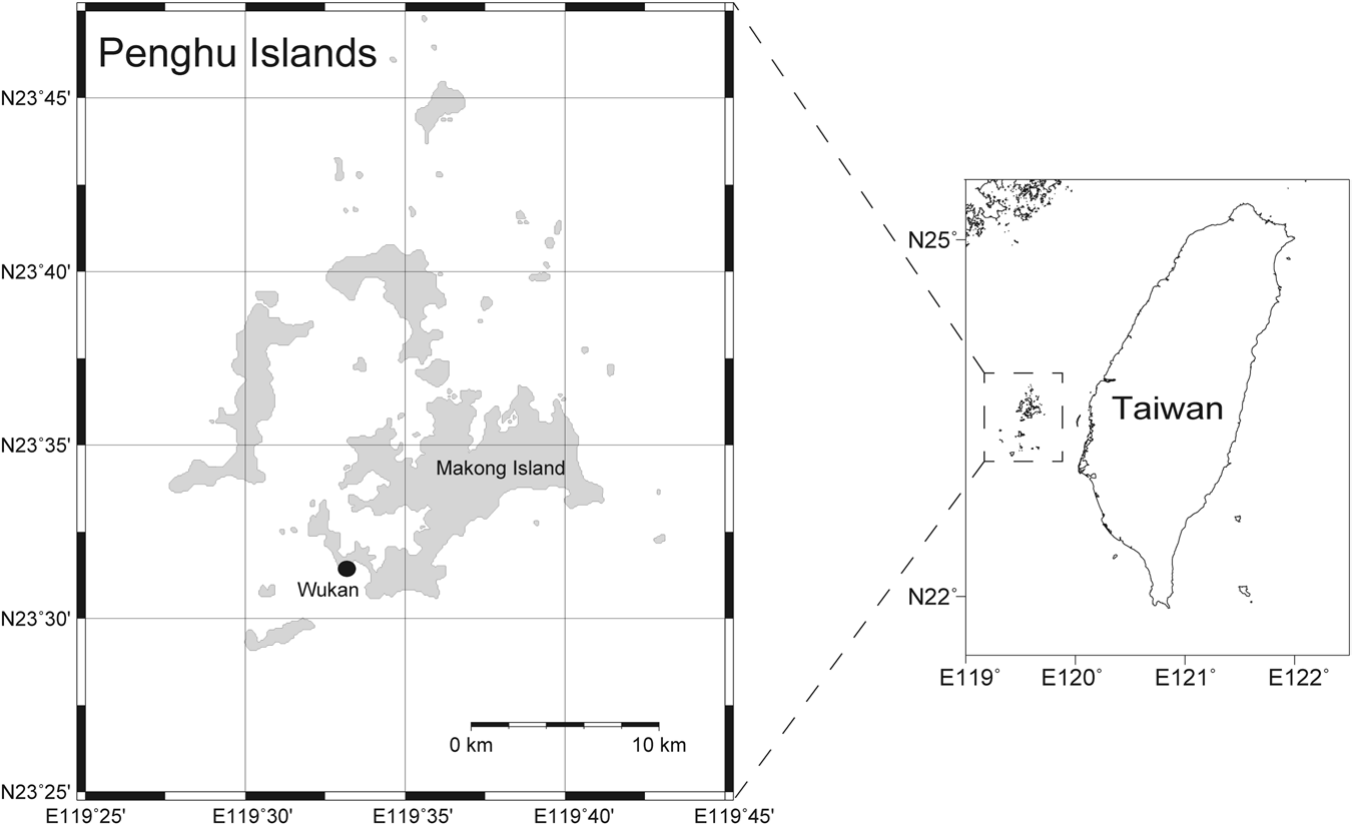
Sampling locations in Wukan (23°32′38.9″N, 119°37′32.3″E). *Acropora muricata* were collected in June 2012 and February 2013. This map was generated using Generic Mapping Tools (version 5; http://gmt.soest.hawaii.edu).

To detect short-term dynamics in coral-associated bacterial communities exposed to various temperatures and to compare this variation between summer and winter, *Acropora muricata* coral colonies were collected from the Penghu Archipelago, in western Taiwan (Fig. 1). Nubbins from *Acropora muricata* were exposed to 6 acute temperature treatments, 10 °C, 15 °C, 20 °C, 25 °C (in winter or 26 °C in summer), 30 °C, and 33 °C in both summer and winter. Changes in coral-associated bacteria were determined after 12, 24 and 48 h (Table 1).

**Table 1:** Temperatures and times of coral and seawater samples collected from seawater tanks in Penghu.

Season	Sampling time (h)	Temperature (°C)					
		10	15	20	26	30	33
**Summer June 2012**	**0**				SC		
	**12**	S110	S115	S120	S126	S130	S133
	**24**	S210	S215	S220	S226	S230	S233
	**48**	S410	S415^*^	S420	S426	S430	S433
**Winter Feb 2013**		**10**	**15**	**20**	**25**	**30**	**33**
	**0**			WC			
	**12**	W110	W115	W120	W125	W130	W133
	**24**	W210	W215	W220	W225	W230	W233
	**48**	W410	W415	W420	W425	W430	W433

Nubbins of coral sample (n = 3) and 1 L seawater sample (n = 1) were collected from each treatment, acclimation (0 h) and experimental seawater tanks after 12, 24, and 48 h treatment. Abbreviations in sample names: The first character is sampling season, S: Summer, W: Winter; the following number is sampling time, 1:12 h, 2:24 h, 4:48 h; and the last 2 numbers are treatment temperature.^*^Only 2 coral nubbins (n = 2) and 1 seawater sample were collected for the bacterial community at this sampling time.

**Figure 2:**
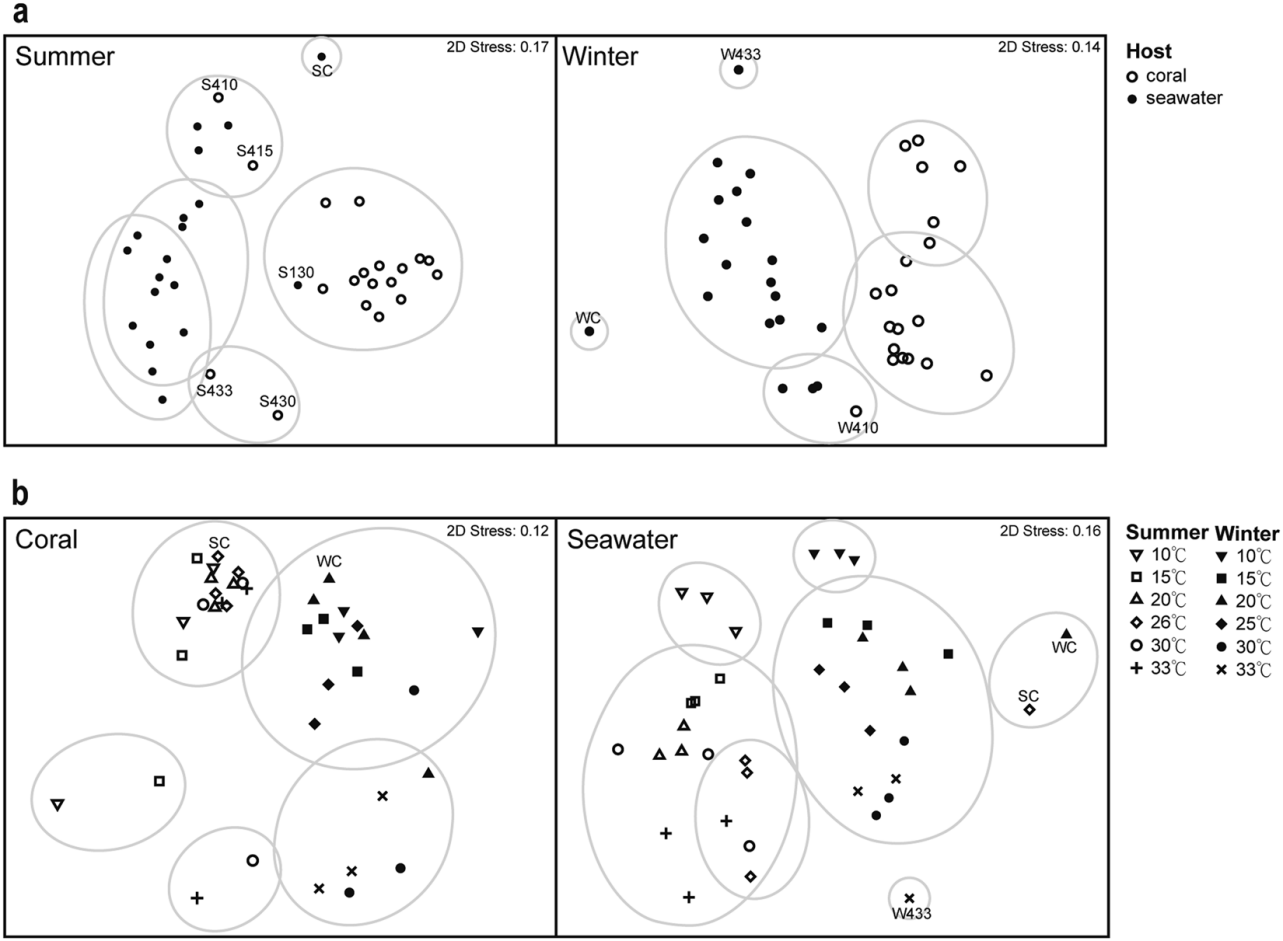
NMDS based on Bray-Curtis similarity matrix with complete linkage among samples. All OTUs abundance in samples were nomalised by numbers of sequences in each sample. The 3 biological replicates of coral sample from each treatment were combined into 1 representative sample. Similarities among samples > 10 are circled by grey lines. (**a**) Bacterial communities varied between coral and seawater samples in both summer (left) and winter (right). (**b**) In both coral (left) and seawater (right) samples, bacterial communities differed between samples collected in summer versus winter.

Analysis of bacterial community composition was based on OTUs with 97% nucleotide sequence identity and matrices of Bray-Curtis similarity. Overall, bacterial communities differed between coral and seawater samples, both in summer (2-way crossed ANOSIM, R = 0.653, P < 0.001) and winter (2-way crossed ANOSIM, R = 0.769, P < 0.001; Fig. 2a). Community compositions also differed between samples collected in summer and winter (Fig. 2b). In coral samples, variation was more obvious between seasons (2-way nested ANOSIM, R = 0.904, P = 0.002), compared to that under various temperature treatments (2-way nested ANOSIM, R = 0.23, P = 0.003). Bacterial communities were also different between seasons in seawater samples (2-way crossed ANOSIM, R = 0.965, P < 0.001).

**Figure 3:**
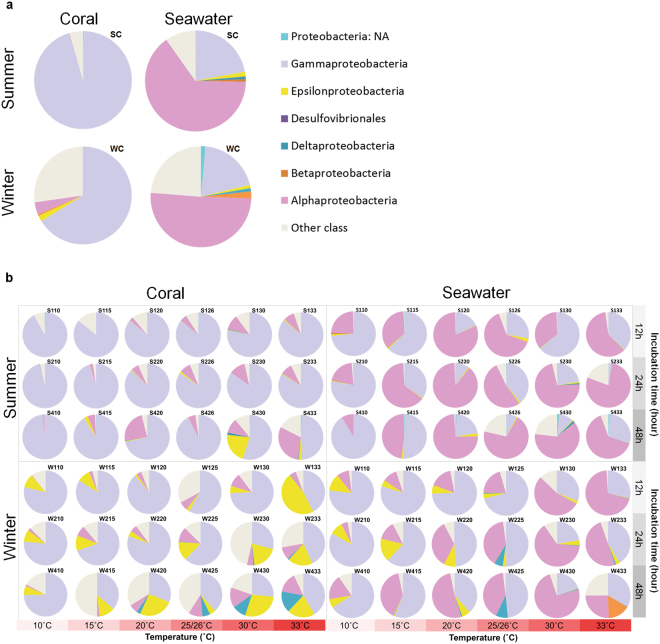
Bacterial composition (class level) in samples. (**a**) Before treatment (0 h), *Gammaproteobacteria* were dominant in coral samples and dominant *Alphaproteobacteria* in seawater samples in both summer and winter. (**b**) Changes in the bacterial community under temperature stress from 12 to 48 h. For coral samples in winter, *Epsilonproteobacteria* and *Deltaproteobacteria* increased under heat stress.

To facilitate detection of differences in bacterial community composition, OTUs assigned to the same bacterial class were combined and presented in a pie chart. Regarding class-level changes in bacterial composition, for samples before temperature treatment, *Gammaproteobacteria* was dominant (95.2 and 66.2% of relative abundance) in coral samples in both seasons, whereas *Alphaproteobacteria* was dominant (64.7 and 50.7%) in seawater samples (Fig. 3a). More *Alphaproteobacteria* and *Epsilonproteobacteria* (4.1 and 1.9%) were detected in winter than in summer samples. However, when coral samples were under thermal treatment (Fig. 3b), *Epsilonproteobacteria* and *Deltaproteobacteria* increased, particularly in winter samples with heat stress (10.3% after 48 h at 30 °C and 15.9% after 48 h at 33 °C), whereas after 48 h of heat stress, *Gammaproteobacteria* decreased in all coral samples from both seasons (minimum relative abundance = 26.5%).

### Diversity indices of bacterial communities

**Figure 4:**
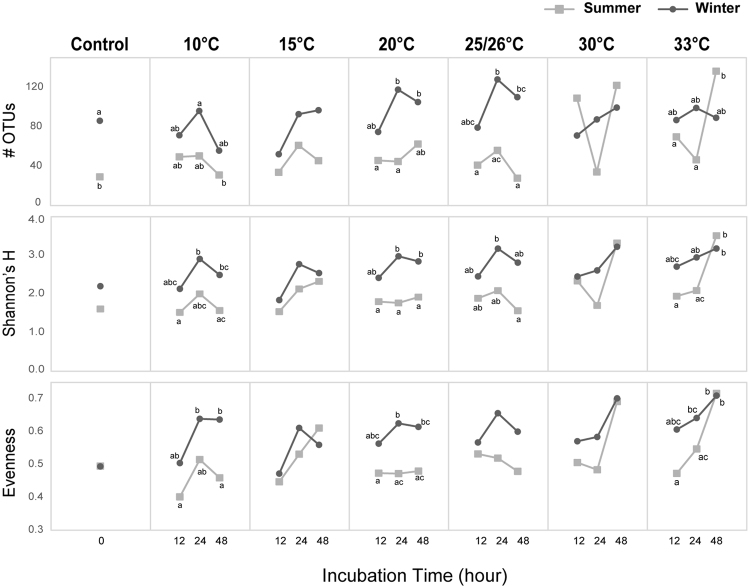
Average of alpha-diversity in bacteria communities over time in 2 seasons. Squares and light grey represent summer samples, whereas circles and dark grey represent winter samples. Differences (*P* < 0.05) among samples are indicated by different letters in each panels. Averages and error bars of triplicate samples are in Supplementary Figure S1.

Before treatment (0 h), coral samples nearly always had lower diversity, evenness or richness than seawater samples in both seasons (Supplementary Table S1a). Winter coral samples had greater richness and diversity than summer coral samples. However, after heat treatment, diversity in summer coral samples was highest among all samples (Fig. 4, Supplementary Fig. S1 and Table S1b).

Diversity fluctuated among sampling times during treatment. Under heat treatment (30 and 33 °C), OTU number of summer coral samples decreased between 12 and 24 h, but rapidly increased after 24 h. Furthermore, diversity of samples under cold treatment (10 °C) had a different dynamic pattern, as it increased between 12 and 24 h of treatment, but decreased after 24 h. In general, dynamics of alpha-diversity in coral-associated bacterial communities had distinctively different patterns between heat and cold stresses.

### Influence factors for the variation of bacterial composition in coral samples

**Figure 5:**
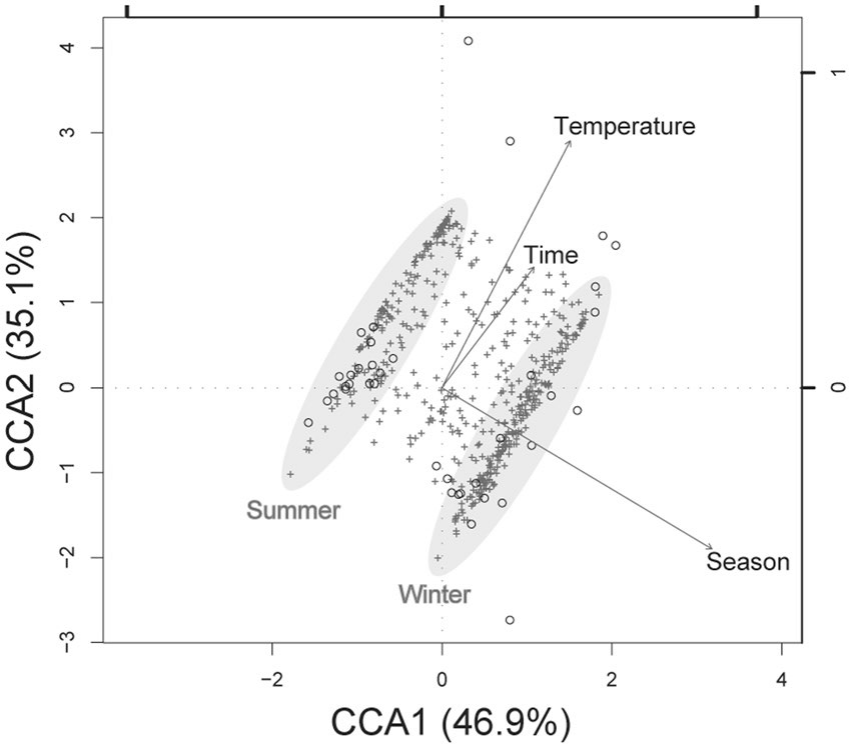
CCA analysis for effects of 3 major factors (temperature, time and season) on distribution of bacteria in coral samples. All OTUs (crosses) in coral samples (circles) are included and distribution of OTUs represents differences between seasons. The scales of the 3 factors are shown in top and right axes. Light grey ellipses indicate most OTUs distributed in each season.

Three factors were crucial in this study, namely season, temperature and duration of treatment. To determine which of these factors were associated with variation in bacterial composition, canonical correspondence analysis (CCA; Fig. 5) was performed. Eigen values of constrained axes for season, temperature and time were 0.499, 0.233 and 0.197, respectively. Therefore, season was most closely associated with bacterial composition of coral samples, with the majority of OTUs clearly separated into summer or winter groups.

### Transition of bacterial taxa in coral samples under thermal stress

**Figure 6:**
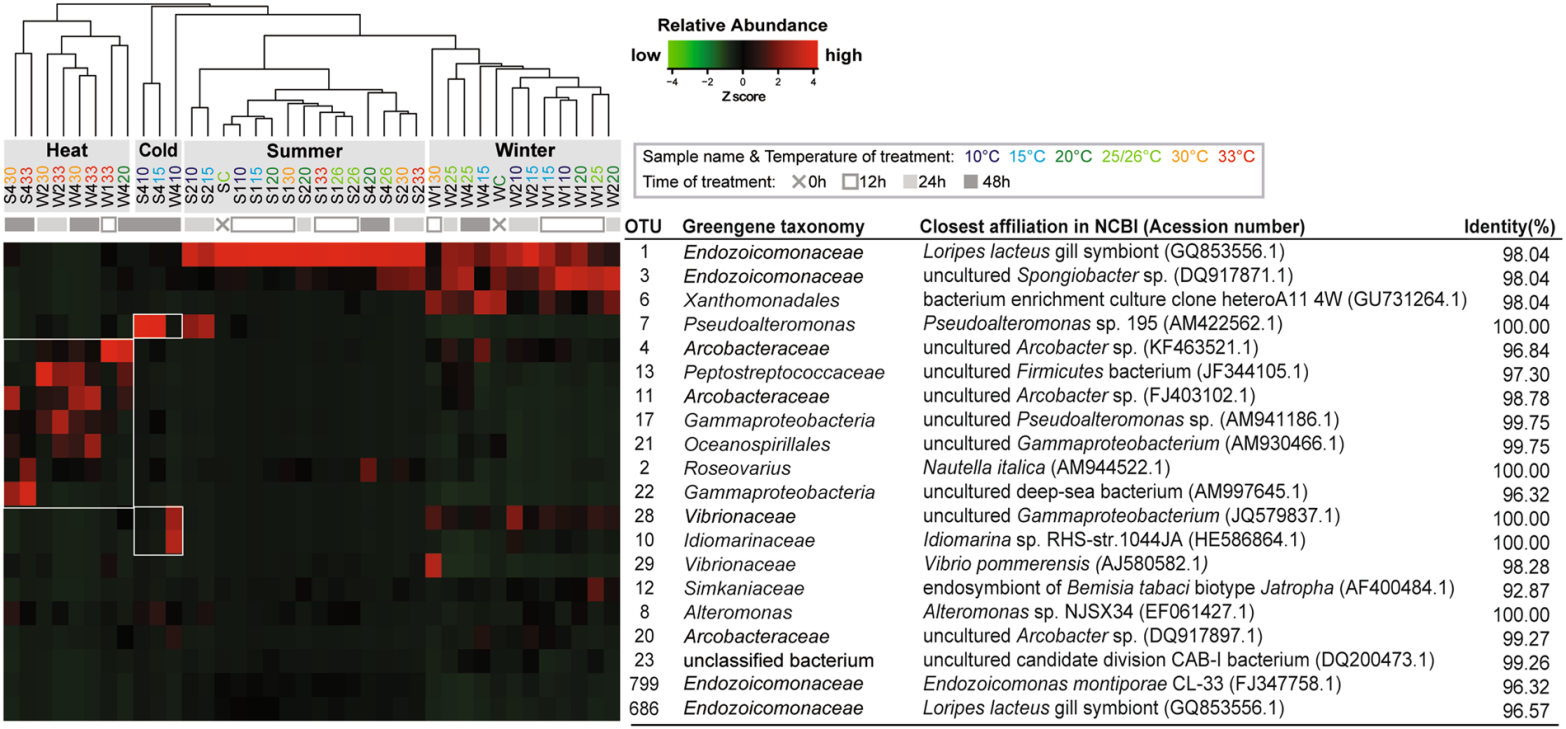
Heatmap of top 20 OTUs in coral samples with Bray-Curtis similarity clustering. The top 20 OTUs in the y-axis had >1% accumulative relative abundance in all coral samples. Coral samples in x-axis were clustered based on Bray-Curtis similarity between all OTUs. Coral samples were separated into 4 groups: “Winter”, “Summer”, “Cold”, and “Heat”. Relative abundance of the top 20 OTUs in samples was presented (after transformation with a z-score). Red represents higher abundance, whereas green indicates relative rare abundance in each sample. Sample names in x-axis with different colors indicate temperature of treatment, and duration of treatments in each sample are presented below sample names.

To evaluate dominant OTUs, a heatmap was prepared to display relative abundance of the top 20 OTUs in all coral samples and their taxonomic affiliations, based on Greengenes database and the blast result of NCBI (Fig. 6). Our Bray-Curtis similarity results showed the presence of 3 clusters, which corresponded to samples with a similar bacterial community as control samples, samples under heat stress (“Heat” group in Fig. 6), and samples under cold stress (“Cold” group in Fig. 6), respectively. Samples within a cluster had Bray-Curtis similarity >20, except the 3 samples under cold stress. Moreover, the cluster, including control samples, was further separated into 2 sub-clusters, each with Bray-Curtis similarity >40, that corresponded to samples collected in summer and in winter (“Summer” and “Winter” groups in Fig. 6). Therefore, coral samples were divided into 4 groups: “Summer”, “Winter”, “Cold”, and “Heat”.

For the “Summer” and “Winter” clusters, composition of bacterial community was dominated by OTU1, affiliated with the bacteria *Candidatus* Endozoicomonaceae in the Greengenes database and belonging to the genus *Endozoicomonas*, Family *Hahellaceae* in SILVA and RDP databases. However, there were more dominant OTUs detected in the winter cluster, OTU3 and OTU6, affiliated with *Endozoicomonaceae* and *Xanthomonadales*, respectively.

The “Cold” cluster was comprised of 2 summer samples (S410 and S415) and 1 winter sample (W410). The 2 summer samples were dominated by OTU7 (*Pseudoalteromonas*), whereas the winter sample was dominated by 2 OTUs, OTU10 (*Idiomarinaceae*) and OTU28 (*Vibrionaceae*). Unlike other clusters, this cluster was not fully supported by Bray-Curtis similarity, and no common OTU was detected between summer and winter samples. OTU1 and OTU3 (*Endozoicomonaceae*) shifted to OTU7 (*Pseudoalteromonas*) in summer samples. A clear transition of bacterial communities was observed after 24 h treatment at either 15 or 10 °C. Samples S215 and S210 were co-dominated by OTU1 and OTU7. With more prolonged incubation (48 h), OTU1 decreased and summer samples were solely dominated by OTU7. A similar dynamic pattern was also present in winter samples incubated at 10 °C for 24 or 48 h. Co-occurrence of the dominant OTU1, OTU3 (*Endozoicomonaceae*) and OTU28 (*Idiomarinanceae*) in sample W210 (at 10 °C for 24 h) shifted to OTU28 and OTU10 (*Vibronaceae*) that were dominant in sample W410 (10 °C for 48 h).

The last “Heat” cluster was composed of 6 winter samples and only 2 summer samples. The latter were under heat stress at 30 or 33 °C for 48 h (S430, S433). In contrast, the bacterial community of the winter samples in this cluster changed quickly after 12 h at 33 °C (W133). In the beginning of the shift, the dominant bacterial profile changed from OTU1, OTU3 and OTU6 to OTU4 (*Arcobacteraceae*), followed by OTU13 (*Peptostreptococcaceae*) that predominated at 24 h, whereas OTU11 (*Arcobacteraceae*) became dominant after 48 h of incubation at 30 or 33 °C.

### Temporal changes in 4 key bacteria at various temperatures

Four dominant bacterial taxa were highly correlated with various temperature treatments (Fig. 6), including *Endozoicomonaceae* (OTU1, 3, 799, 686), *Alteromonadales* (OTU7, 8, 10, 17), *Vibrionaceae* (OTU28, 29), and *Arcobacteraceae* (OTU4, 11, 20). To characterize dynamics of these 4 bacterial taxa during treatment, xyplots were generated (Supplementary Fig. S3).

**Figure 7:**
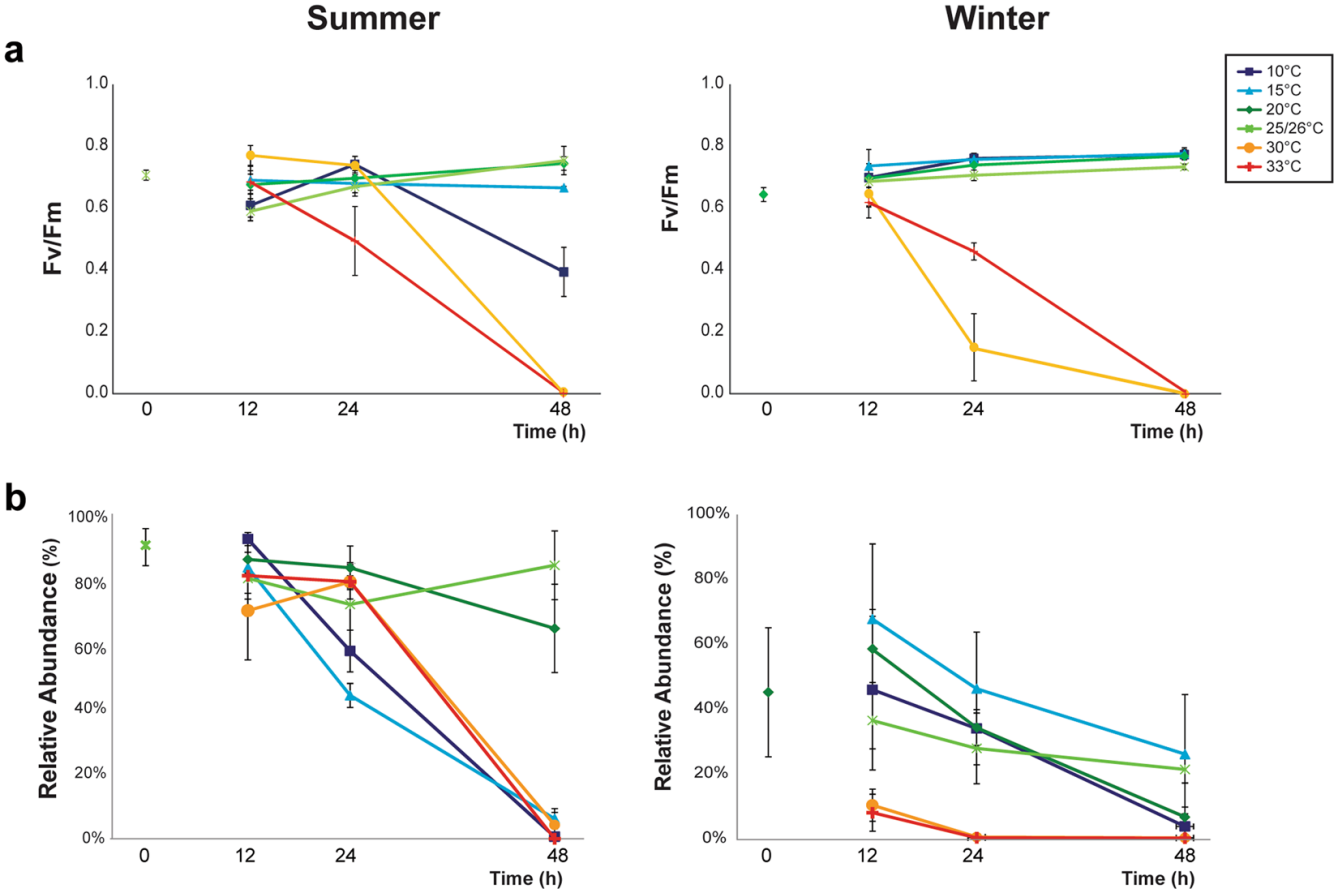
Xyplot for (**a**) photosynthetic efficiency of *Symbiodinium* and (**b**) relative abundance of *Endozoicomonaceae* in samples under various temperature treatments over time. Average and standard error of the mean of 3 biological replicates are in the y axis, whereas the x axis represents time after treatment.

Relative abundance of *Endozoicomonaceae* in summer samples exceeded 80% (Supplementary Fig. S3 and Fig. 7b), and was higher than that in winter samples (only 40% in samples before treatment). Abundance in summer decreased to nearly 0% under heat or cold stress (30 and 33 °C for heat stress and 10 and 15 °C for cold stress) for 48 h, whereas those abundances were >60% at 20 and 25 °C. The rate of decrease was faster under cold treatment than heat treatment in summer samples. In winter samples, the highest relative abundance of *Endozoicomonaceae* was in samples at 15 °C (68% at 12 h and 26% at 48 h) rather than in samples at 20 or 26 °C. For winter samples under heat treatment >30 °C, relative abundances rapidly decreased to 0% with 24 h incubation, which was faster than winter samples under cold treatment (near 0% at 10 °C for 48 h). This pattern was opposite to summer samples under temperature treatment.

Compared to *Endozoicomonaceae*, abundance of *Alteromonadales*, *Arcobacteraceae*, and *Vibrionaceae* was near 0% in samples before treatment (summer or winter; Supplementary Fig. S3). However, *Alteromonadales* in summer samples increased to >60% under cold stress (10 or 15 °C) at 48 h, whereas *Endozoicomonaceae* decreased to near 0% after the same incubation. In winter samples, abundance of *Alteromonadales* did not increase as high as those in summer, but still reached 15% relative abundance under 10 °C with 48 h incubation.

For *Vibrionaceae*, abundances were very low (<3%) in all summer samples, but increased quickly (up to 35%) within 12 h in winter samples under heat treatment at 30 °C. In addition, under cold stress (10 °C), abundance of *Vibrionaceae* was maintained at 15% after 24 h (Supplementary Fig. S3).

Relative abundance of *Arcobacteraceae* that belonged to *Epsilonproteobacteria* in winter samples, like *Vibrionaceae*, greatly increased (up to 50%) within 12 h under heat stress (33 °C). After 48 h treatment in winter samples, relative abundance was maintained at >15% in all samples except those at 25 °C, whereas those of *Vibrionaceae* under heat stress in winter samples were low and decreased after 24 h. However, *Arcobacteraceae* were low in summer samples (averaged < 3%) except for samples under 30 °C at 48 h (average = 22%). In summary, dynamics of bacterial community composition under temperature stresses with various time treatments were more complex in winter versus summer samples.

### Short-term dynamics of *Symbiodinium* and coral-associated microbes under heat and cold stress

To compare responses of bacteria and *Symbiodinium* in coral under stress, photosynthetic efficiency (Fv/Fm) and density of *Symbiodinium* were determined (Fig. 7a and Supplementary Fig. S4). Under heat treatment, both density and photosynthetic efficiency of *Symbiodinium* were low compared to control (Fv/Fm < 0.2, density < 1000 cells) after 48 h. Photosynthetic efficiency of winter samples was decreased at 24 h under 30 °C; this was sooner than those of the summer samples. On the contrary, under cold treatment, both parameters of *Symbiodinium* in samples at 10 and 15 °C treatments remained stable, except photosynthetic efficiency of *Symbiodinium* decreased in summer samples at 10 °C with 48 h incubation (Fig. 6a).

## Discussion

This study compared changes between the 2 seasons in *Acropora muricata*-associated bacterial communities under an array of temperature stresses. Bacterial communities collected in the 2 seasons had distinct patterns. Shifts in the bacterial community under cold stress were slower in winter than in summer, whereas a more rapid shift occurred under heat stress in both seasons. Compared to previous research, the present study had the lowest similarity of bacterial communities between samples of winter and summer (<10 Bray-Curtis similarity using cluster with complete linkage in Fig. 2, or <40 using cluster with average linkage in Fig. 6). To our knowledge, there are only 5 published studies comparing coral-associated bacterial communities between seasons. Two reports had results similar to ours^33,37^, whereas the other 3 detected limited differences between seasons^31,34,38^. Apparent inconsistencies among reports for compositional shifts in bacterial community between seasons were likely due to multiple factors, including differences in environments, time, coral species, or methods. Of those factors, environmental variation may have had the biggest impact. In this study, the waters around Penghu are dominated by varying currents between seasons, resulting in a much larger seasonal range in temperature than reefs in other reports, including those in the Mediterranean Sea, the Great Barrier Reef and South Taiwan (tropical region).

According to the meteorological database, cold waters of the China Coastal Current coming from high latitudes cause low temperatures at the Penghu sampling site in winter (the lowest historical temperature was 12.1 °C; Supplementary Fig. S5) compared to Cheng-Kung Township on the east coast of Taiwan (the lowest recorded temperature was 19.9 °C in December of 2009). Based on this study, the low temperature likely affected the bacterial community in *A*. *muricata*, although more evidence should be collected. The bacterial group *Xanthomonadales* predominated in winter samples (Fig. 6), but not in summer samples, and contributed to major variations between seasons. This bacterial group was also abundant in the region of a deep coral, *Lophelia pertusa* at a high-latitude area, the SE Rockall Bank in the NE Atlantic (55°28′55.2″N 15°48′19.8″W), with a water temperature <10 °C at the sampling site^39^ (van Bleijswijk *et al*., 2015).

Our study is 1 of only 2 recent studies to determine dynamics of bacterial community in corals under heat stress <1 d in duration. Ziegler and coworkers (2017)^30^ used *Acropora hyacinthus* samples from back-reef pools of Ofu Island of the U.S. National Park of American Samoa, and demonstrated the coral-associated bacterial community responded rapidly (within 20 h) to heat stress. In the present study, the response of bacterial community in *Acropora muricata* samples was also rapid and varied between seasons, within 12 h in winter and 48 h in summer under acute heat stress (Fig. 6 and Supplementary Fig. S3).

There were 4 dominant and sensitive bacterial groups for early responses to temperature stresses (Supplementary Fig. S3), namely *Endozoicomonaceae*, *Alteromonadales*, *Vibrionaceae*, and *Arcobacteracea*, in terms of changes in relative abundance. Those 4 bacterial groups were likely associated with changes in coral holobiont status under stress.

*Endozoicomonaceae* is a prominent group, likely associated with coral health^4,40–42^, and also a common marine invertebrate-associated bacterium, present in various reefs worldwide^43^. In our study, coral-associated *Endozoicimonaceae* were sensitive to thermal stress. The relative abundance of these bacteria significantly decreased under either heat (30 and 33 °C) or cold stress (10 and 15 °C), consistent with previous research. For instance, relative abundance of *Endozoicomonas* has been reported to largely decrease in response to abiotic stresses, e.g. temperature increases^44^, ocean acidification^45^, or anthropogenic impacts (sedimentation and sewage)^46^. Furthermore, bleaching of *Acropora* corals in the Great Barrier Reef caused substantial disappearance of *Endozoicomonas* ^11^. Collectively, based on present results and previous reports, we inferred that rapid changes in *Endozoicomonas* could be a robust bioindicator for changes in the coral holobiont.

*Alteromonadales* was associated with stressed or diseased corals^47^ and was regarded as an indicator of aged mucus of coral or mucus in disturbed coral^4^. Interestingly, *Pseudoalteromonas*, belonging to *Alteromonadales*, inhibited *Vibrio* ^3,48^ and had antibacterial activity in the coral holobiont^49^. There was a high relative abundance of *Pseudoalteromonas* in summer samples under cold stress (OTU7 in S410, S415, S210 and S215 samples; Fig. 6) and concurrently, low relative abundance of *Vibrio* (OTU28 and 29) in these 4 summer samples.

*Vibrio* bacteria are highly associated with corals under stress^50^. Moreover, increased *Vibrio* was often reported when *Acropora* spp. were under stress^46,51^, especially heat stress^11,44^. In the present study, relative abundance of *Vibrionaceae* in winter increased not only under heat stress but also under cold stress (Supplementary Fig. S3). Although *Vibrio* have been widely reported to be highly associated with coral bleaching or temperature stress^11^, their relative abundance in this study was not as high as other pathogens (i.e., *Arcobacteraceae*; Supplementary Fig. S3).

All 3 OTUs that belonged to *Arcobacteraceae* in the top 20 dominant OTUs (OTU4, 11, and 20) were all assigned to the genus *Arcobacter* in the NCBI database. These 3 OTUs were detected in the winter coral samples with a low relative abundance at the beginning, but they increased at subsequent sampling times. In the present study, *Arcobacter* was a stress-associated bacterial group and quickly grew in coral under thermal stress. *Arcobacter* have been reported in association with coral diseases, including White Syndrome and Brown Band Disease in *Acropora muricata* in the Great Barrier Reef^47^ and White Plague Disease in *Montastraea faveolata* in the Caribbean Sea^52^. Perhaps *Arcobacter* is an opportunistic bacterium in coral rather than a specific pathogen.

The link between stability of a bacterial community and adjustable heat tolerance after acclimation was recently proposed^29,30^. The present study also supports a similar link between seasonal acclimation and thermal tolerance range in bacterial community. As bacteria were under cold stress (15 and 10 °C), shifting of dominant OTUs occurred at 10 °C in winter, lower than that in summer (15 °C) (Fig. 6). With 15 °C treatment, the relative abundance of *Endozoicomonaceae* was highest among all other treatments in winter, but these bacteria decreased to near undetectable levels in summer (Fig. 7b). Therefore, bacteria in winter samples more readily acclimated themselves to a colder temperature than those in summer samples. Similarly, seasonal acclimation was also detected under heat stress. Shifting of dominant bacteria was more quickly in winter samples than in the summer samples under heat stress (Fig. 6). In winter, the bacterial community changed quickly into the “Heat” group (heat stress) when exposed to 33 °C for just 12 h, whereas summer samples were grouped into the “Heat” group only after treatment for 48 h. In addition, increased relative abundance of *Arcobacter* (stress-associated bacteria) was slower in summer than winter (48 versus 12 h; Supplementary Fig. S3).

Notably, there was a high relative abundance of *Endozoicomonaceae* between 15 and 30 °C (Fig. 7b and Supplementary Fig. S3). This temperature range was different from the optimal growth temperature of *Endozoicomonas* spp., reported as 25 to 30 °C^53–56^. Above 30 °C, relative abundance of *Endozoicomonaceae* rapidly decreased in this study (Fig. 7b). However, these bacteria in winter samples were still largely detected at a temperature (15 °C for 48 h) colder than the lowest optimal temperature (25 °C). Therefore, we hypothesize that there was some cold acclimation in this bacterial group (OTU1 and OTU3 in Fig. 6). The inconsistency of the temperature ranges might have been caused by species variation and perhaps other factors, including cold acclimation. It was noteworthy that *Endozoicomonaceae* were also widely detected in cold habitats. Although there is no information about these bacteria in hexacorals under cold stress, *Endozoicomonaceae* dominates in azooxanthellate octocorals in cold-water environments^31,57^. For example, *Endozoicomonaceae* were detected in a deep-sea coral gorgonian *Anthothela* sp., collected at 400-600 m, where seawater temperatures were 5.5 to 7.1 °C^58^. High relative abundance of *Endozoicomonaceae* through various seasons was also reported in another cold-water gorgonian *Eunicella verrucosa* collected at 15-27 m along the south-west coast of England with a seawater temperature 13.9 to 17.0 °C^34^. Collectively, it was apparent that *Endozoicomonaceae* have potential to acclimate to a cold environment.

In this study, *Acropora muricata-*associated bacteria in Penghu had different tolerance ranges to thermal stress between summer and winter after seasonal acclimation (Supplementary Fig. S5). We further inferred that the response and tolerance ranges of coral-associated bacteria were associated with short-term “memory” in thermal history. Tolerance ranges in the 2 seasons were estimated to be >15 °C to <30 °C in summer, and >10 °C to <30 °C in winter. To better define the tolerance ranges for *Acropora*-associated bacterial community in Penghu, finer scale of temperature treatments is needed. Regardless, we concluded that the tolerance range may cover 15 to 25 °C in winter and 20 to 26 °C in summer, as the bacterial community remained similar to the control sample in the same cluster (Fig. 6 and Supplementary Fig. S5a).

This estimated temperature range based on community stability differed from average temperatures in summer and winter in the thermal history of the sampling site (Supplementary Fig. S5a)^59^. The upper limit of the estimated tolerance range for coral under heat stress in summer was 26 °C, which was lower than the average temperature in summer in Penghu (27.1 °C from the averages of June, July, and August). On the contrary, the lower limit of the estimated tolerance range for coral under cold stress was 15 °C, lower than the average temperature in winter (February, March, and April), 18.8 °C. Therefore, both ranges did not match to one another (Supplementary Fig. S5a).

However, the estimated temperature ranges of bacterial community aligned well with the seawater temperature profile 3 mo before the sampling was done, in either winter or summer (orange or blue square in Supplementary Fig. S5b), suggesting that their response was more likely to associate with the temperature history within the previous 3 mo, rather than the entire thermal history (i.e. from 12.1 to 30.2 °C). This apparent short-term acclimation was also supported by another study^30^, where the ability of thermal tolerance disappeared 1 y after corals were transplanted from a high-temperature region to lower temperature region. We inferred that the short-term “memory” of temperature in a coral holobiont was important, at least for stabilization of the bacterial community.

We modified our hypothesis that the tolerance temperature range for the bacterial community is adjustable in different seasons, based on acclimation to thermal history within the short-term (previous 3 mo in this study). In future studies, smaller differences in temperature and interval, and more prolonged stress treatments should provide a clearer picture of the thermal tolerance range for coral-associated bacteria.

## Conclusions

In this study, variations of microbial communities associated with corals under both heat and cold stress for up to 2 d were characterized in 2 seasons, summer and winter. This was apparently the first study to document responses of the coral-associated bacterial community to cold stress. In this study, the bacterial community in *Acropora* from Penghu varied according to season. Variations affected the response and tolerance range of stable homeostasis in coral-associated bacteria under temperature stress. The tolerance range of coral-associated bacteria under various temperatures was related to short-term thermal history (3 mo before stress). Finally, observed shifts in the abundance and diversity of bacterial communities were further considered in the context of known interactions between the coral holobiont and associated bacteria.

## Materials and Methods

### Study site, sample collection and experimental design

Summer and winter experiments were conducted in June 2012 and February 2013, respectively, in sub-tropical, Penghu, Taiwan (Fig. 1 and Table 1). The map in Fig. 1 was generated using the Generic Mapping Tools (GMT v.5; http://gmt.soest.hawaii.edu). Branches from 5 colonies of *Acropora muricata* were collected from Wukan (23°32′38.9″N, 119°37′32.3″East). Branches from each colony were used to make 2–3 cm nubbins that were attached (with epoxy) to 2-ml Eppendorf tubes. All nubbins were maintained in a 26 °C (summer) or 20 °C (winter) acclimation tank for 2–3 d prior to the onset of experiment period. After acclimation, 3 coral nubbins each from 5 colonies and 50 mL seawater were sampled, whereas remaining nubbins were distributed into 6 experimental seawater tanks (15 × 15 × 15 cm, ~3 l capacity) maintained at temperatures of 10, 15, 20, 26, 30 or 33 °C respectively. Each tank was placed in a bigger tank (that was used as a water bath). Circulation inside experimental tanks was a closed system, with 2/3 of the water replaced with filtered (0.2 μm) seawater every 24 h. Temperature of the water bath was maintained by Hailea 150 A aquarium chillers (set to 26 and 20 °C in summer and winter, respectively). Each of the 6 experimental tanks contained small aquarium heater rods to maintain temperatures. Each tank had at least 60 nubbins (12 nubbins from each of 5 colonies), with 3 nubbins from different colonies randomly sampled after 12, 24, and 48 h, respectively. A total of 57 nubbins each during summer and winter experiment were sampled and fixed in absolute ethanol and 19 seawater samples each during summer and winter experiment were collected and filtered immediately through cellulose acetate membranes with 0.2-μm pores (Adventec, Tokyo, Japan), transported at 4 °C and stored at −20 °C prior to DNA extraction.

### *Symbiodinium* density and photosynthetic efficiency

To calculate density of *Symbiodinium* per unit coral surface area, coral surface areas were measured using an aluminum foil method^60^. *Symbiodinium in hospite* were removed from coral branches using an airbrush with filtered seawater (0.2-µm mesh filter) and fixed in 4% formaldehyde. The solution of *Symbiodinium* and coral tissue was centrifuged (4000 × *g* for 10 min) to separate coral tissue from *Symbiodinium* cells. Fresh-filtered seawater was added to the resulting pellet, mixed thoroughly and centrifuged (4000 × *g* for 10 min). For counting *Symbiodinium* cells, 3 aliquots (1 ml each) were used and cells were counted (hemocytometer and light microscope) 3 times for each aliquot. The average number of cells was converted to *Symbiodinium* density normalized to coral surface area (cm^2^).

The dark-adapted maximum quantum yield of photosystem II (Fv/Fm) was measured using a Walz^®^ Junior-pulse amplitude-modulated (PAM) flurometer with a 0.8 s saturating pulse of >4500 µmol photons m^−2^s^−1^ and gain value of 12. Dark-adapted yield values (F_v_/F_m_) were determined from 5 coral nubbins from each colony, collected 2 h after sunset. Analysis of variance (ANOVA) was used to detect variations among coral samples.

### Total DNA extraction, amplification of V6-V8 region in bacterial 16S rRNA gene, and tagging PCR

Coral nubbins were washed with TE buffer (10 mM Tris-HCl, 1 mM EDTA, pH 8), frozen in liquid nitrogen, and homogenized using a sterile mortar and pestle. Coral powder and seawater samples were transferred into TE buffer for total DNA extraction using a modified CTAB method^61,62^.

Amplification of the 16S ribosomal RNA gene was performed by PCR using a pair of universal bacterial primers: 968 F (5′-AACGCGAAGAACCTTAC-3′) and Uni1391R (5′-ACGGGCGGTGWGTRC-3′) specifically designed for the bacterial V6-V8 hypervariable region^42,63,64^. The PCR was performed in 50-μL reaction volumes, consisting of 1.5 U *TaKaRa Ex Taq* (Takara Bio, Otsu, Japan), 1X *TaKaRa Ex Taq* buffer, 0.2 mM deoxynucleotide triphosphate mixture (dNTP), 0.2 mM of each primer, and 50 to 150 ng purified total DNA. The thermocycler was set to an initial step of 94 °C for 5 min, 30 cycles of 94 °C for 30 s, 52 °C for 20 s and 72 °C for 45 s, and a final extension at 72 °C for 10 min. Target DNA bands (~420 bp) were electrophoresed on a 1.5% agarose gel, and specific bands were eluted using a QIAEX II Gel Extraction Kit (Qiagen, Valencia, CA, USA).

To tag each bacterial V6-V8 amplicon with a unique barcode sequence, tag primers were designed with 4 overhanging nucleotides at 5′ ends of common primers. The tagging reaction was performed with a 5-cycle PCR, with a reaction program of 94 °C for 30 s, 52 °C for 20 s, and 72 °C for 45 s with the modified primers. Amplicons were purified by the same gel elution method described above. Concentration of DNA was determined with a Qubit dsDNA HS assay (Invitrogen, Carlsbad, CA, USA).

### Illumina MiSeq pair-end sequencing and data processing

Summer and winter V6-V8 amplicons were pooled into 2 independent libraries for 2 × 250 pair-end reads in Illumina MiSeq sequencing (Yourgene Bioscience, Taipei, Taiwan). Overall, 581,860 and 567,874 raw reads were obtained after pair-end reads were merged. After sorting and trimming, high-quality reads were extracted using MOTHUR^65^ with the following criteria: (1) read lengths between 380 and 450 bps; (2) average quality score >27; (3) homopolymer length <8 bp; and (4) removal of reads with any ambiguous base (N). Thereafter, the 4 nucleotide tags and primer sequences were removed. Chimeric reads were inspected and eliminated by UCHIME^66^ with USEARCH v7.0.1090 (parameters: reference mode, rdp_gold database, and mindiv of 5). Qualified sequences were retained for subsequent analyses.

For operational taxonomic units (OTU) analysis, qualified reads were pooled and assigned OTUs with a cutoff value at 97% identity by the UPARSE pipeline^67^. Each OTU was classified with a bootstrap value set to 0.8 using RDP classifier^68^ implemented in MOTHUR. The alignment template was the SILVA reference database (release 102). Sequences were annotated with taxonomies in the Greengenes database. There were 114 OTUs that belonged to unclassified bacteria, according to the database of Greengenes taxonomy. These OTUs were further examined and blasted with the RDP Seqmatch tool. In subsequent analyses, 27 OTUs affiliated to bacteria with >0.8 ab_score were retained, whereas the remaining 87 OTUs were removed.

### Data analyses

Diversity indices of microbial communities were presented without singleton OTU after the UPARSE pipeline, except for calculation of richness. Diversity indices of Shannon-Weaver and Gini-Simpson, values of richness and evenness were calculated using MOTHUR software. Average and standard error (3 biological replicates) of alpha-diversity were calculated. Two-way analysis of variance and Tukey’s HSD comparisons were conducted to detect significant differences in diversity among sampling times and between seasons.

Three biological replicates from each treatment were combined into one representative sample and relative abundances of each classified bacterial OTU in individual samples were calculated by dividing the sum of sequence numbers in the sample^37^. For composition of bacterial community in each sample, OTUs assigned to the same class were combined, and relative abundances of each class in an individual sample were presented in a pie chart. It is noteworthy that primer bias from different regions of the 16S rRNA gene may cause variation in analysis of relative abundance^69^.

To analyze beta-diversity and determine relationships of bacterial communities between samples, relative abundances of OTUs in individual samples were incorporated into a matrix to estimate a distance matrix (Bray-Curtis distance). Then, the result was presented in a non-Metric multi-Dimensional Scaling (nMDS) analysis with complete linkage using Primer 6 software (PRIMER-E, Lutton, Ivybridge, UK), a plot of Canonical Correspondence Assay (CCA), and a heatmap with hierarchical clustering (CLUSTER) by average linkage using an R program (https://www.r-project.org/).

In the nMDS analysis, differences in bacterial community composition among samples were tested by ANOSIM analysis. Differences among bacterial communities in coral samples from various temperatures were tested within seasons, using 2-way nested ANOSIM analysis, whereas differences among bacterial communities in seawater samples from different temperatures and seasons were determined using 2-way crossed ANOSIM.

The 20 most abundant OTUs were selected for both CCA and heatmap analyses. In the heatmap, relative abundance data of the 20 OTUs were presented after transformation to z-scores, and their taxonomies identified and listed. Clustering of coral samples along the x-axis was based on Bray-Curtis similarity matrix with average linkage using relative abundance of all OTUs in each coral sample, although only the relative abundances of the top 20 OTUs were shown.

### Xyplot for *Endozoicomonaceae, Alteromonadels, Vibrionaceae*, and *Arcobacteraceae*

Average and standard error (3 biological replicates) of relative abundances of sequences of *Endozoicomonaceae*, *Alteromonadels*, *Vibrionaceae*, *and Arcobacteraceae* were calculated. Analysis of variance (ANOVA) was used to determine significance of variation among coral samples.

### Data Accessibility

Multiplex sequenced reads (bacterial 16S V6-V8 region) were deposited in the NCBI Sequence Read Archive (accession number PRJNA379752).

## Electronic supplementary material

Supplementary Information
