# Supplementary material for: Dynamics of coral-associated bacterial communities acclimated to temperature stress based on recent thermal history
Source: Sci Rep. 2017 Nov 2;7:14933. doi: 10.1038/s41598-017-14927-3 (PMC5668310; doi:10.1038/s41598-017-14927-3)
Supplement: Supplementary file 1 — Supplementary Information [file 41598_2017_14927_MOESM1_ESM.doc]

**Dynamics of coral-associated bacterial communities acclimated to temperature stress based on recent thermal history**

Jia-Ho Shiu^1,2,3^, Shashank Keshavmurthy^2^, Pei-Wen Chiang^2^, Hsing-Ju Chen^2^, Shueh-Ping Lou^2^, Ching-Hung Tseng^4^, Hernyi Justin Hsieh^5^, Chaolun Allen Chen^2^, Sen-Lin Tang^1,2,6,*^

*^1^Molecular and Biological Agricultural Sciences Program, Taiwan International Graduate Program, Academia Sinica, Taipei, Taiwan, and National Chung-Hsing University, Taichung, Taiwan; ^2^Biodiversity Research Center, Academia Sinica, Taipei, Taiwan; ^3^Graduate Institute of Biotechnology, National Chung-Hsing University, Taichung, Taiwan; ^4^Germark Biotechnology Co., Ltd., Taichung 40767, Taiwan; ^5^Penghu Marine Biology Research Center, Fishery Research Institute, Council of Agriculture, Magong, Penghu 880, Taiwan, ROC; ^6^Biotechnology Center, National Chung-Hsing University, Taichung, Taiwan;*

*E-mail: sltang@gate.sinica.edu.tw

Telephone: +886-2-27893863

Facsimile: +886-2-27890844

Address: Biodiversity Research Center, Academia Sinica, No. 128 Sec. 2, Academia Rd., Nankang, Taipei 11529, Taiwan

**Supplementary Tables and Figures**

**Supplementary Table S1.** Sequence information and diversity estimates as represented in V6-V8 regions of the 16S rRNA gene. All coral and seawater samples are listed and seawater samples are highlighted with a grey background. (**a**) Samples before treatment, 0 hour (0 h). (**b**) Samples under various temperature treatments at 3 sampling times (12, 24 and 48 h).

**a**

| **Season** | **Temp.** | **0 h** | | | | | | |
| --- | --- | --- | --- | --- | --- | --- | --- | --- |
|  |  | **Sample** | ***N*^b^** | ***S*^c^** | **Richness^d^** | **Evenness^e^** | **Shannon** | **Simpson^f^** |
| **Summer** | **26** | **SCa** | 869 | 20 | 2.38 | 0.51 | 1.54 | 0.68 |
|  |  | **SCb** | 10596 | 43 | 3.98 | 0.36 | 1.36 | 0.66 |
|  |  | **SCc** | 902 | 24 | 2.37 | 0.61 | 1.94 | 0.82 |
|  |  | **SCS** | 4080 | 213 | 11.63 | 0.69 | 3.71 | 0.93 |
| **Winter** | **20** | **WCa** | 3712 | 74 | 8.68 | 0.43 | 1.85 | 0.71 |
|  |  | **WCb** | 2917 | 78 | 8.08 | 0.48 | 2.09 | 0.81 |
|  |  | **WCc** | 2690 | 106 | 11.95 | 0.57 | 2.68 | 0.86 |
|  |  | **WCS** | 3894 | 182 | 6.13 | 0.76 | 3.98 | 0.96 |

b

| **Season** | **Temp.** | **12 h** | | | | | | |  | **24 h** | | | | | | | | | | | | | |  | | **48 h** | | | | | | | | | | | | | | |
| --- | --- | --- | --- | --- | --- | --- | --- | --- | --- | --- | --- | --- | --- | --- | --- | --- | --- | --- | --- | --- | --- | --- | --- | --- | --- | --- | --- | --- | --- | --- | --- | --- | --- | --- | --- | --- | --- | --- | --- | --- |
|  |  | **Sample** | ***N*^b^** | ***S*^c^** | **Richness^d^** | **Evenness^e^** | **Shannon** | **Simpson^f^** |  | **Sample** | ***N*^b^** | | ***S*^c^** | | **Richness^d^** | | **Evenness^e^** | | **Shannon** | | **Simpson^f^** | |  | | **Sample** | | ***N*^b^** | | ***S*^c^** | | **Richness^d^** | | **Evenness^e^** | | **Shannon** | | **Simpson^f^** | |  | |
| **Summer** | **10** | **S110a** | 11622 | 45 | 3.44 | 0.34 | 1.28 | 0.62 |  | **S210a** | 2223 | | 33 | | 2.99 | | 0.50 | | 1.74 | | 0.76 | |  | | **PS410a** | | 1762 | | 30 | | 3.08 | | 0.46 | | 1.57 | | 0.67 | |  | |
|  |  | **S110b** | 9138 | 74 | 7.83 | 0.39 | 1.70 | 0.74 |  | **S210b** | 3385 | | 60 | | 7.08 | | 0.50 | | 2.06 | | 0.81 | |  | | **PS410b** | | 1914 | | 34 | | 3.66 | | 0.44 | | 1.56 | | 0.67 | |  | |
|  |  | **S110c** | 2214 | 29 | 3.29 | 0.47 | 1.59 | 0.71 |  | **S210c** | 2467 | | 58 | | 9.14 | | 0.54 | | 2.21 | | 0.83 | |  | | **PS410c** | | 2223 | | 29 | | 2.69 | | 0.47 | | 1.60 | | 0.68 | |  | |
|  |  | **S110S** | 1693 | 129 | 15.18 | 0.59 | 2.87 | 0.81 |  | **S210S** | 5029 | | 166 | | 15.13 | | 0.56 | | 2.85 | | 0.87 | |  | | **PS410S** | | 1935 | | 47 | | 4.56 | | 0.50 | | 1.92 | | 0.70 | |  | |
|  | **15** | **S115a** | 14459 | 43 | 3.85 | 0.29 | 1.09 | 0.57 |  | **S215a** | 975 | | 42 | | 6.02 | | 0.55 | | 2.06 | | 0.80 | |  | | **PS415a** | | 1926 | | 52 | | 4.87 | | 0.62 | | 2.43 | | 0.84 | |  | |
|  |  | **S115b** | 1040 | 34 | 3.65 | 0.57 | 1.99 | 0.80 |  | **S215b** | 1410 | | 47 | | 7.30 | | 0.58 | | 2.23 | | 0.84 | |  | | **PS415b** | | N.A. | | N.A. | | N.A. | | N.A. | | N.A. | | N.A. | |  | |
|  |  | **S115c** | 2693 | 24 | 2.62 | 0.49 | 1.55 | 0.74 |  | **S215c** | 9477 | | 94 | | 9.05 | | 0.46 | | 2.11 | | 0.81 | |  | | **PS415c** | | 1226 | | 39 | | 4.53 | | 0.61 | | 2.22 | | 0.82 | |  | |
|  |  | **S115S** | 3186 | 151 | 17.13 | 0.50 | 2.52 | 0.83 |  | **S215S** | 4370 | | 120 | | 11.26 | | 0.40 | | 1.90 | | 0.67 | |  | | **PS415S** | | 2087 | | 68 | | 8.44 | | 0.44 | | 1.85 | | 0.72 | |  | |
|  | **20** | **S120a** | 2149 | 37 | 4.50 | 0.46 | 1.65 | 0.72 |  | **S220a** | 1247 | | 28 | | 4.52 | | 0.48 | | 1.60 | | 0.74 | |  | | **PS420a** | | 2358 | | 79 | | 9.79 | | 0.50 | | 2.16 | | 0.80 | |  | |
|  |  | **S120b** | 1990 | 57 | 8.49 | 0.46 | 1.84 | 0.76 |  | **S220b** | 1753 | | 42 | | 8.32 | | 0.44 | | 1.65 | | 0.73 | |  | | **PS420b** | | 1723 | | 24 | | 2.78 | | 0.47 | | 1.49 | | 0.67 | |  | |
|  |  | **S120c** | 1984 | 43 | 5.46 | 0.51 | 1.92 | 0.78 |  | **S220c** | 2124 | | 64 | | 8.42 | | 0.50 | | 2.07 | | 0.78 | |  | | **PS420c** | | 2575 | | 84 | | 11.14 | | 0.48 | | 2.10 | | 0.79 | |  | |
|  |  | **S120S** | 4473 | 98 | 10.68 | 0.32 | 1.46 | 0.51 |  | **S220S** | 3941 | | 85 | | 10.01 | | 0.26 | | 1.15 | | 0.36 | |  | | **PS420S** | | 2263 | | 56 | | 6.86 | | 0.41 | | 1.65 | | 0.58 | |  | |
|  | **26** | **S126a** | 2103 | 30 | 3.31 | 0.49 | 1.67 | 0.75 |  | **S226a** | 1411 | | 58 | | 7.30 | | 0.62 | | 2.51 | | 0.86 | |  | | **PS426a** | | 1397 | | 42 | | 5.09 | | 0.54 | | 2.01 | | 0.77 | |  | |
|  |  | **S126b** | 1260 | 18 | 1.29 | 0.54 | 1.57 | 0.72 |  | **S226b** | 1885 | | 27 | | 2.75 | | 0.35 | | 1.15 | | 0.59 | |  | | **PS426b** | | 1673 | | 24 | | 4.03 | | 0.41 | | 1.31 | | 0.67 | |  | |
|  |  | **S126c** | 2146 | 75 | 10.51 | 0.56 | 2.43 | 0.86 |  | **S226c** | 2371 | | 83 | | 11.56 | | 0.59 | | 2.61 | | 0.86 | |  | | **PS426c** | | 989 | | 17 | | 2.34 | | 0.49 | | 1.38 | | 0.65 | |  | |
|  |  | **S126S** | 4467 | 84 | 10.96 | 0.50 | 2.23 | 0.85 |  | **S226S** | 2402 | | 54 | | 9.17 | | 0.51 | | 2.03 | | 0.82 | |  | | **S426S** | | 4332 | | 73 | | 8.25 | | 0.42 | | 1.81 | | 0.74 | |  | |
|  | **30** | **S130a** | 3428 | 210 | 15.84 | 0.63 | 3.37 | 0.91 |  | **S230a** | 1358 | | 39 | | 5.75 | | 0.46 | | 1.69 | | 0.73 | |  | | **S430a** | | 2037 | | 102 | | 10.88 | | 0.66 | | 3.05 | | 0.90 | |  | |
|  |  | **S130b** | 2052 | 72 | 6.94 | 0.56 | 2.39 | 0.82 |  | **S230b** | 791 | | 29 | | 3.80 | | 0.51 | | 1.71 | | 0.73 | |  | | **S430b** | | 2807 | | 130 | | 10.15 | | 0.72 | | 3.51 | | 0.95 | |  | |
|  |  | **S130c** | 2859 | 45 | 6.94 | 0.33 | 1.24 | 0.59 |  | **S230c** | 1397 | | 34 | | 4.77 | | 0.48 | | 1.71 | | 0.69 | |  | | **S430c** | | 3169 | | 134 | | 11.14 | | 0.69 | | 3.39 | | 0.93 | |  | |
|  |  | **S130S** | 2358 | 76 | 11.27 | 0.45 | 1.97 | 0.78 |  | **S230S** | 2360 | | 87 | | 9.19 | | 0.55 | | 2.43 | | 0.78 | |  | | **S430S** | | 5703 | | 132 | | 9.05 | | 0.61 | | 2.97 | | 0.90 | |  | |
|  | **33** | **S133a** | 6363 | 126 | 11.31 | 0.51 | 2.45 | 0.80 |  | **S233a** | 2486 | | 57 | | 5.89 | | 0.56 | | 2.27 | | 0.85 | |  | | **S433a** | | 2106 | | 124 | | 8.43 | | 0.73 | | 3.54 | | 0.93 | |  | |
|  |  | **S133b** | 2085 | 47 | 6.33 | 0.43 | 1.67 | 0.71 |  | **S233b** | 961 | | 39 | | 4.36 | | 0.62 | | 2.26 | | 0.84 | |  | | **S433b** | | 4463 | | 161 | | 12.06 | | 0.70 | | 3.54 | | 0.93 | |  | |
|  |  | **S133c** | 2145 | 36 | 4.20 | 0.48 | 1.72 | 0.71 |  | **S233c** | 1674 | | 43 | | 7.45 | | 0.46 | | 1.74 | | 0.72 | |  | | **S433c** | | 1965 | | 124 | | 11.24 | | 0.72 | | 3.46 | | 0.94 | |  | |
|  |  | **S133S** | 1541 | 59 | 7.53 | 0.59 | 2.40 | 0.87 |  | **S233S** | 3668 | | 60 | | 8.42 | | 0.41 | | 1.66 | | 0.73 | |  | | **S433S** | | 2082 | | 84 | | 8.44 | | 0.50 | | 2.23 | | 0.80 | |  | |
| **Winter** | **10** | **W110a** | 2703 | 72 | 7.58 | 0.57 | 2.45 | 0.84 |  | **W210a** | 4518 | | 102 | | 4.65 | | 0.69 | | 3.19 | | 0.93 | |  | | **W410a** | | 313 | | 24 | | 4.01 | | 0.62 | | 1.97 | | 0.79 | |  | |
|  |  | **W110b** | 10438 | 104 | 7.71 | 0.53 | 2.46 | 0.84 |  | **W210b** | 3547 | | 76 | | 8.17 | | 0.65 | | 2.81 | | 0.90 | |  | | **W410b** | | 3718 | | 68 | | 5.04 | | 0.66 | | 2.80 | | 0.90 | |  | |
|  |  | **W110c** | 4382 | 38 | 4.94 | 0.41 | 1.49 | 0.71 |  | **W210c** | 3463 | | 110 | | 13.00 | | 0.58 | | 2.72 | | 0.88 | |  | | **W410c** | | 2945 | | 75 | | 5.19 | | 0.63 | | 2.71 | | 0.89 | |  | |
|  |  | **W110S** | 1877 | 98 | 12.83 | 0.57 | 2.60 | 0.84 |  | **W210S** | 2681 | | 87 | | 9.33 | | 0.54 | | 2.42 | | 0.84 | |  | | **W410S** | | 887 | | 51 | | 4.75 | | 0.58 | | 2.28 | | 0.77 | |  | |
|  | **15** | **W115a** | 5741 | 99 | 8.25 | 0.63 | 2.89 | 0.92 |  | **W215a** | 2351 | | 101 | | 9.49 | | 0.64 | | 2.97 | | 0.91 | |  | | **W415a** | | 5658 | | 116 | | 10.39 | | 0.50 | | 2.37 | | 0.80 | |  | |
|  |  | **W115b** | 1616 | 20 | 1.87 | 0.33 | 1.00 | 0.56 |  | **W215b** | 3704 | | 110 | | 7.85 | | 0.69 | | 3.25 | | 0.94 | |  | | **W415b** | | 1743 | | 70 | | 8.33 | | 0.56 | | 2.36 | | 0.83 | |  | |
|  |  | **W115c** | 1573 | 37 | 3.75 | 0.46 | 1.65 | 0.66 |  | **W215c** | 2702 | | 67 | | 6.70 | | 0.50 | | 2.10 | | 0.78 | |  | | **W415c** | | 3427 | | 104 | | 6.79 | | 0.62 | | 2.90 | | 0.86 | |  | |
|  |  | **W115S** | 2234 | 122 | 14.33 | 0.65 | 3.13 | 0.91 |  | **W215S** | 1027 | | 67 | | 7.97 | | 0.67 | | 2.81 | | 0.90 | |  | | **W415S** | | 1615 | | 69 | | 7.48 | | 0.55 | | 2.33 | | 0.81 | |  | |
|  | **20** | **W120a** | 1666 | 94 | 11.80 | 0.59 | 2.69 | 0.85 |  | **W220a** | 1960 | | 111 | | 11.85 | | 0.72 | | 3.40 | | 0.94 | |  | | **W420a** | | 2725 | | 75 | | 6.11 | | 0.63 | | 2.70 | | 0.88 | |  | |
|  |  | **W120b** | 2078 | 71 | 9.04 | 0.50 | 2.11 | 0.80 |  | **W220b** | 4458 | | 125 | | 11.78 | | 0.55 | | 2.65 | | 0.84 | |  | | **W420b** | | 1901 | | 127 | | 17.08 | | 0.65 | | 3.13 | | 0.88 | |  | |
|  |  | **W120c** | 1433 | 59 | 7.92 | 0.60 | 2.46 | 0.87 |  | **W220c** | 2756 | | 117 | | 11.63 | | 0.61 | | 2.88 | | 0.88 | |  | | **W420c** | | 1861 | | 113 | | 9.79 | | 0.57 | | 2.71 | | 0.78 | |  | |
|  |  | **W120S** | 834 | 72 | 11.64 | 0.64 | 2.75 | 0.88 |  | **W220S** | 1237 | | 84 | | 8.73 | | 0.70 | | 3.11 | | 0.92 | |  | | **W420S** | | 965 | | 83 | | 9.38 | | 0.77 | | 3.39 | | 0.95 | |  | |
|  | **25** | **W125a** | 2946 | 69 | 6.63 | 0.54 | 2.27 | 0.85 |  | **W225a** | 4455 | | 147 | | 11.51 | | 0.63 | | 3.16 | | 0.91 | |  | | **W425a** | | 2065 | | 122 | | 11.16 | | 0.54 | | 2.57 | | 0.73 | |  | |
|  |  | **W125b** | 1760 | 56 | 7.40 | 0.57 | 2.29 | 0.84 |  | **W225b** | 2991 | | 109 | | 7.48 | | 0.71 | | 3.32 | | 0.93 | |  | | **W425b** | | 1092 | | 103 | | 10.53 | | 0.79 | | 3.66 | | 0.96 | |  | |
|  |  | **W125c** | 3414 | 112 | 10.47 | 0.60 | 2.81 | 0.87 |  | **W225c** | 4114 | | 128 | | 9.68 | | 0.63 | | 3.05 | | 0.90 | |  | | **W425c** | | 2246 | | 105 | | 13.13 | | 0.48 | | 2.21 | | 0.67 | |  | |
|  |  | **W125S** | 1623 | 95 | 11.53 | 0.62 | 2.83 | 0.87 |  | **W225S** | 1212 | | 81 | | 10.05 | | 0.64 | | 2.80 | | 0.87 | |  | | **W425S** | | 1164 | | 83 | | 11.09 | | 0.71 | | 3.13 | | 0.92 | |  | |
|  | **30** | **W130a** | 2363 | 83 | 9.19 | 0.63 | 2.80 | 0.87 |  | **W230a** | 5443 | | 85 | | 3.75 | | 0.61 | | 2.72 | | 0.85 | |  | | **W430a** | | 2027 | | 109 | | 11.79 | | 0.73 | | 3.41 | | 0.95 | |  | |
|  |  | **W130b** | 1715 | 90 | 9.89 | 0.73 | 3.26 | 0.93 |  | **W230b** | 3486 | | 83 | | 7.06 | | 0.53 | | 2.36 | | 0.79 | |  | | **W430b** | | 2418 | | 93 | | 7.09 | | 0.69 | | 3.15 | | 0.93 | |  | |
|  |  | **W130c** | 1538 | 40 | 3.77 | 0.35 | 1.31 | 0.54 |  | **W230c** | 4814 | | 94 | | 7.33 | | 0.61 | | 2.76 | | 0.88 | |  | | **W430c** | | 1221 | | 96 | | 11.02 | | 0.68 | | 3.12 | | 0.91 | |  | |
|  |  | **W130S** | 3119 | 88 | 9.73 | 0.61 | 2.72 | 0.88 |  | **W230S** | 2253 | | 66 | | 5.67 | | 0.67 | | 2.81 | | 0.90 | |  | | **W430S** | | 2380 | | 100 | | 10.07 | | 0.66 | | 3.04 | | 0.89 | |  | |
|  | **33** | **W133a** | 4372 | 82 | 5.49 | 0.56 | 2.46 | 0.80 |  | **W233a** | 3589 | | 99 | | 9.28 | | 0.65 | | 2.99 | | 0.91 | |  | | **W433a** | | 721 | | 78 | | 9.10 | | 0.77 | | 3.37 | | 0.94 | |  | |
|  |  | **W133b** | 2434 | 97 | 7.09 | 0.71 | 3.27 | 0.92 |  | **W233b** | 3433 | | 103 | | 9.33 | | 0.61 | | 2.85 | | 0.88 | |  | | **W433b** | | 1276 | | 98 | | 12.24 | | 0.70 | | 3.19 | | 0.91 | |  | |
|  |  | **W133c** | 4874 | 81 | 5.15 | 0.55 | 2.40 | 0.77 |  | **W233c** | 3505 | | 95 | | 7.62 | | 0.66 | | 3.01 | | 0.91 | |  | | **W433c** | | 2519 | | 91 | | 5.88 | | 0.66 | | 2.98 | | 0.89 | |  | |
|  |  | **W133S** | 2200 | 67 | 5.39 | 0.55 | 2.31 | 0.78 |  | **W233S** | 1456 | | 73 | | 6.64 | | 0.74 | | 3.17 | | 0.93 | |  | | **W433S** | | 2841 | | 91 | | 8.69 | | 0.78 | | 3.50 | | 0.94 | |  | |
| ^a^Calculations were based on OTUs formed at an evolutionary distance of ≤0.03 (~97% identity). | | | | | | | | | | | |  | |  | |  | |  | |  | |  | |  | |  | |  | |  | |  | |  | |  | |  | | |
| ^b^*N* =number of sequences | | |  |  |  |  |  |  |  |  |  | |  | |  | |  | |  | |  | |  | |  | |  | |  | |  | |  | |  | |  | |  | |
| ^c^*S* = number of OTUs | | |  |  |  |  |  |  |  |  |  | |  | |  | |  | |  | |  | | | |  | |  | |  | |  | |  | |  | |  | |  | |
| ^d^Richness = (number of singleton OTUs-1)/log_10_*N*. The maximum value is (*N*-1)/log_10_*N*. | | | | | | | | |  |  | |  | |  | |  | |  | |  | |  | |  | |  | |  | |  | |  | |  | |  | |  | |  |
| ^e^Evenness =Shannon/ln(the number of OTUs) | | | | | | | | |  |  | |  | |  | |  | |  | |  | |  | |  | |  | |  | |  | |  | |  | |  | |  | |  |
| ^f^Gini-Simpson | |  |  |  |  |  |  |  |  |  |  | |  | |  | |  | |  | |  | |  | |  | |  | |  | |  | |  | |  | |  | |  | |
| The grey background represents seawater samples | | | | | |  |  |  |  |  |  | |  | |  | |  | |  | |  | |  | |  | |  | |  | |  | |  | |  | |  | | |  |

**Supplementary Table S2.** Three-way analysis of variance (ANOVA) was used to determine significance of variation in: a) photosynthetic efficiency of *Symbiodinium* and b) relative abundance of *Endozoicomonaceae* among coral samples.

| a. Photosynthetic efficiency of *Symbiodinium* | | |  |  |  |  |
| --- | --- | --- | --- | --- | --- | --- |
| Factor | ^a^ Df | Sum of square | Mean square | F value | *P*- value | ^b^Significance |
| Season | 1 | 0.0002 | 0.0002 | 0.11 | 7.37E-01 |  |
| Temperature | 5 | 2.4991 | 0.4998 | 302.68 | < 2E-16 | *** |
| Time | 3 | 0.8697 | 0.2899 | 175.56 | < 2E-16 | *** |
| Season x Temperature | 5 | 0.4101 | 0.0820 | 49.67 | < 2E-16 | *** |
| Season x Time | 3 | 0.1219 | 0.0406 | 24.60 | 3.16E-11 | *** |
| Temperature x Time | 10 | 2.1698 | 0.2170 | 131.40 | < 2E-16 | *** |
| Season x Temperature x Time | 10 | 0.3043 | 0.0304 | 18.43 | < 2E-16 | *** |
| Residuals | 76 | 0.1255 | 0.0017 |  |  |  |
|  |  |  |  |  |  |  |
| b. Relative abundance of *Endozoicomonaceae* | | | |  |  |  |
| Factor | ^a^ Df | Sum of square | Mean square | F value | *P*- value | ^b^Significance |
| Season | 1 | 3.8990 | 3.8990 | 125.64 | < 2E-16 | *** |
| Temperature | 5 | 1.6850 | 0.3370 | 10.86 | 7.01E-08 | *** |
| Time | 3 | 3.1360 | 1.0450 | 33.68 | 6.78E-14 | *** |
| Season x Temperature | 5 | 0.9250 | 0.1850 | 5.96 | 1.07E-04 | *** |
| Season x Time | 3 | 0.4510 | 0.1500 | 4.85 | 3.90E-03 | ** |
| Temperature x Time | 10 | 0.8770 | 0.0880 | 2.83 | 4.98E-03 | ** |
| Season x Temperature x Time | 10 | 0.9520 | 0.0950 | 3.07 | 2.58E-03 | ** |
| Residuals | 75 | 2.3280 | 0.0310 |  |  |  |
| **^a^ Degrees of freedom** |  |  |  |  |  |  |
| **^b^ Significance: < 0.001*** ; < 0.01**; < 0.05*** | | |  |  |  |  |

**Supplementary Table S3.** One-way analysis of variance (ANOVA) was used to determine significance of variation in a) photosynthetic efficiency of *Symbiodinium* and b) relative abundance of *Endozoicomonaceae* among coral samples under various temperature treatments at each sampling time.

| **a.** Photosynthetic efficiency of *Symbiodinium* | | | | |  |  |  |  |
| --- | --- | --- | --- | --- | --- | --- | --- | --- |
| Season | Duration of treatment (h) | Factor | ^a^Df | Sum of square | Mean square | F value | *P*- value | ^b^Significance |
| Summer | 12 | Temperature | 5 | 0.063 | 0.013 | 9.174 | 8.69E-04 | *** |
|  |  | Residuals | 12 | 0.016 | 0.001 |  |  |  |
|  | 24 | Temperature | 5 | 0.125 | 0.025 | 9.404 | 7.78E-04 | *** |
|  |  | Residuals | 12 | 0.032 | 0.003 |  |  |  |
|  | 48 | Temperature | 5 | 1.892 | 0.378 | 253.700 | 9.81E-12 | *** |
|  |  | Residuals | 12 | 0.018 | 0.002 |  |  |  |
| Winter | 12 | Temperature | 5 | 0.026 | 0.005 | 2.259 | 1.15E-01 |  |
|  |  | Residuals | 12 | 0.028 | 0.002 |  |  |  |
|  | 24 | Temperature | 5 | 0.911 | 0.182 | 77.730 | 1.02E-08 | *** |
|  |  | Residuals | 12 | 0.028 | 0.002 |  |  |  |
|  | 48 | Temperature | 5 | 2.332 | 0.466 | 3317.000 | <2E-16 | *** |
|  |  | Residuals | 12 | 0.002 | 0.000 |  |  |  |
| **b.** Relative abundance of *Endozoicomonaceae* | | | | |  |  |  |  |
| Season | Duration of treatment (h) | Factor | ^a^Df | Sum of square | Mean square | F value | *P*- value | ^b^Significant |
| Summer | 12 | Temperature | 5 | 0.080 | 0.016 | 0.639 | 6.75E-01 |  |
|  |  | Residuals | 12 | 0.300 | 0.025 |  |  |  |
|  | 24 | Temperature | 5 | 0.350 | 0.070 | 4.691 | 1.32E-02 | * |
|  |  | Residuals | 12 | 0.179 | 0.015 |  |  |  |
|  | 48 | Temperature | 5 | 2.076 | 0.415 | 24.150 | 1.38E-05 | *** |
|  |  | Residuals | 11 | 0.189 | 0.017 |  |  |  |
| Winter | 12 | Temperature | 5 | 0.907 | 0.181 | 2.616 | 8.00E-02 | . |
|  |  | Residuals | 12 | 0.832 | 0.069 |  |  |  |
|  | 24 | Temperature | 5 | 0.548 | 0.110 | 3.777 | 2.75E-02 | * |
|  |  | Residuals | 12 | 0.348 | 0.029 |  |  |  |
|  | 48 | Temperature | 5 | 0.188 | 0.038 | 2.025 | 1.47E-01 |  |
|  |  | Residuals | 12 | 0.223 | 0.019 |  |  |  |
| **^a^ Degrees of freedom** | |  |  |  |  |  |  |  |
| **^b^ Significance: < 0.001*** ; < 0.01**; < 0.05*** | | | | |  |  |  |  |

**Figure S1**


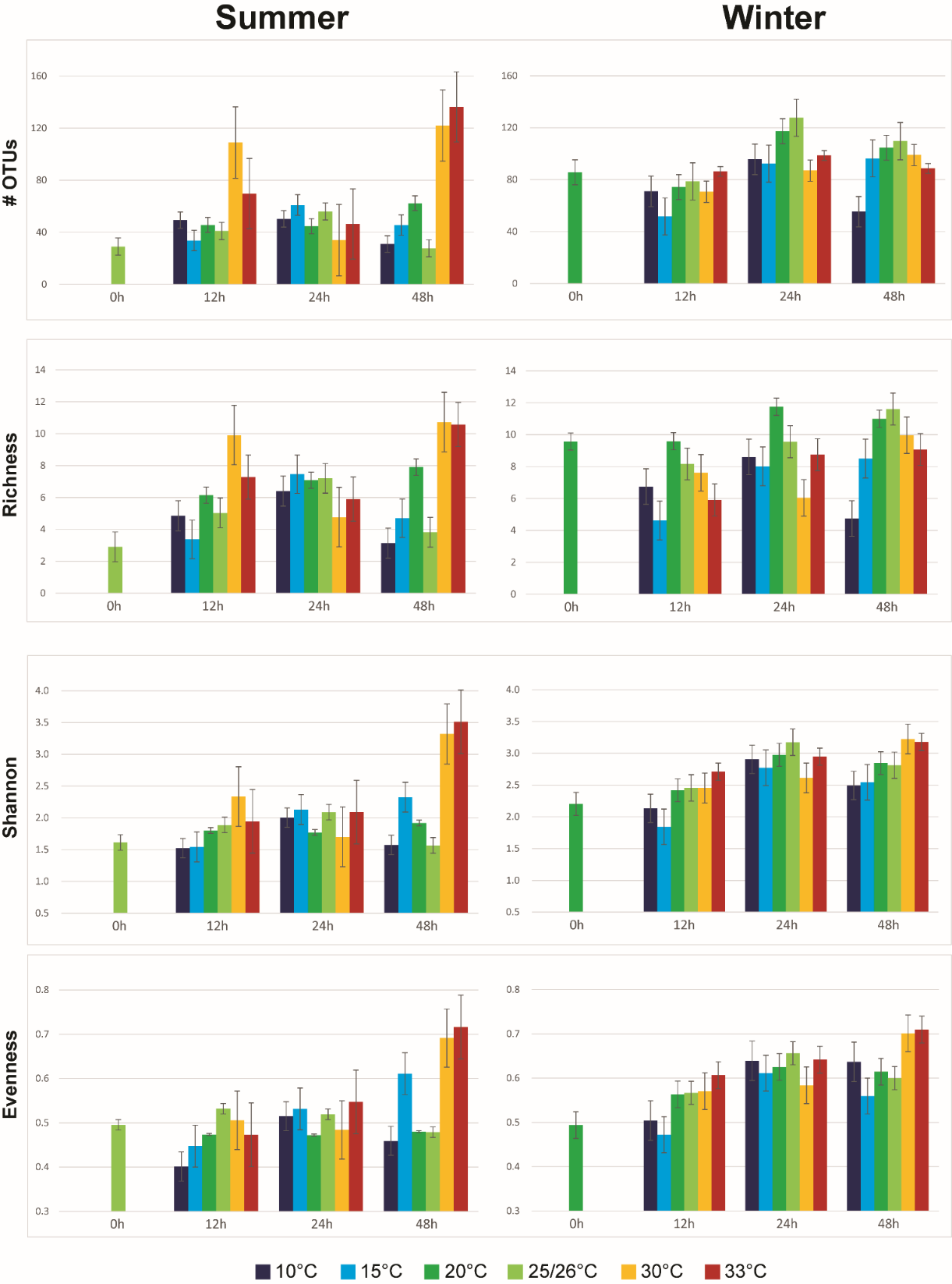


**Supplementary Figure S1.** Richness and Alpha-diversity, expressed as Shannon’s H, of bacterial communities associated with coral samples in response to temperature treatments over time. Error bars represent standard error of mean (triplicates).

**Figure S2**

**
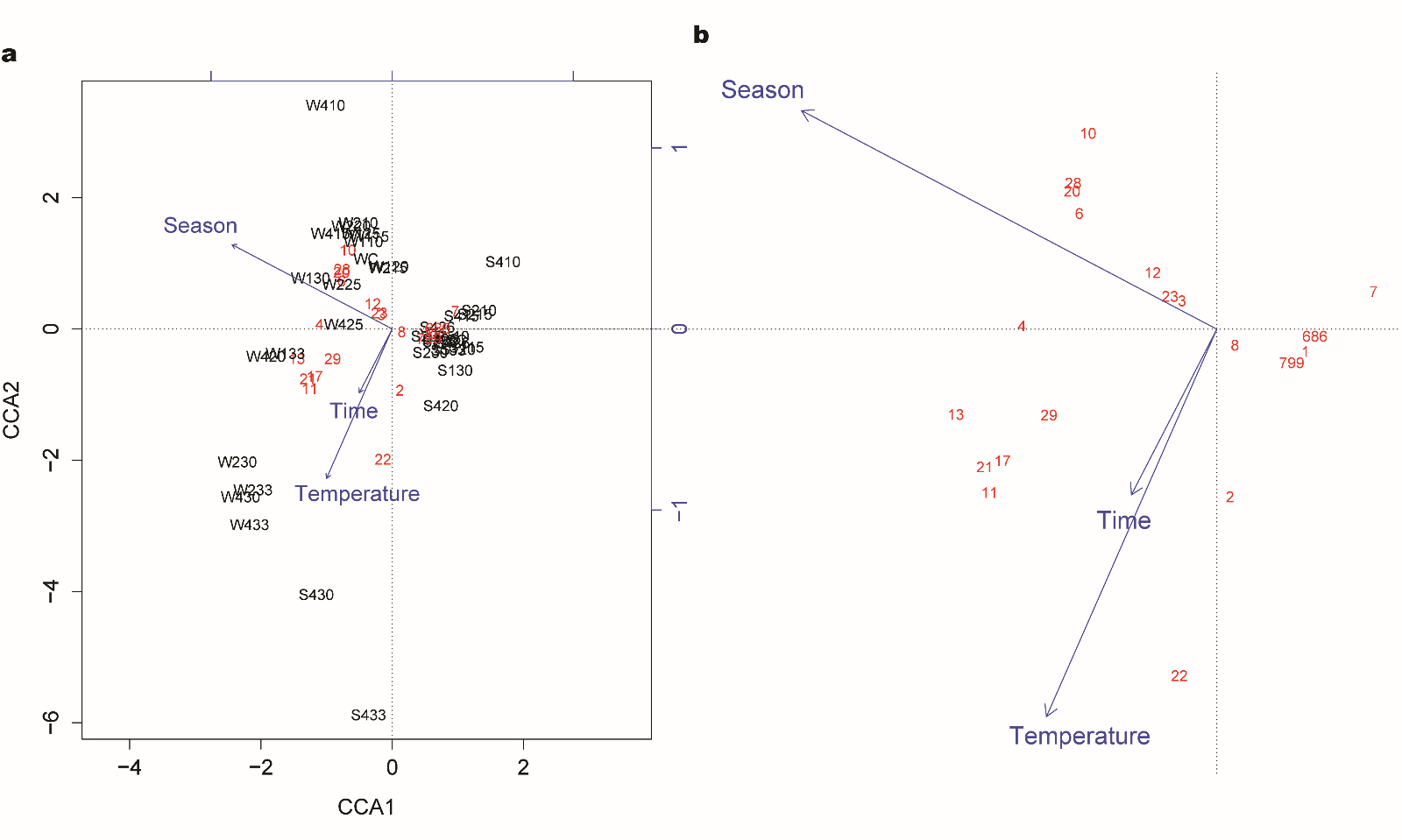
**

**Supplementary Figure S2. CCA analysis for effects of 3 major factors on distribution of bacteria in coral samples**. (a) Only the 20 most abundant OTUs are listed. The scales of 3 factors are shown in top and right axes (in blue). (b) For clarity, we displayed only the 20 OTUs and enlarged the 3 axes. Associations of coral samples with temperature differed between seasons. In winter samples, those OTUs were clearly separated by temperature treatment (Figure S2a), and OTU10 were highly abundant in winter samples under cold treatment, whereas OTU4, 11, 13, 17, 21 and 29 were increased in heat treatment (Figure S2b). Unlike winter samples, most summer samples were grouped with samples before treatment (SC), which included higher relative abundances of *Endozoicomonaceae* (OTU1, 686 and 799). Some OTUs, e.g. OTU22 and 2, in summer samples were positively associated with heat treatment after the longest exposure (48 h), whereas OTU7 was highly associated with cold treatment.

**Figure S3**


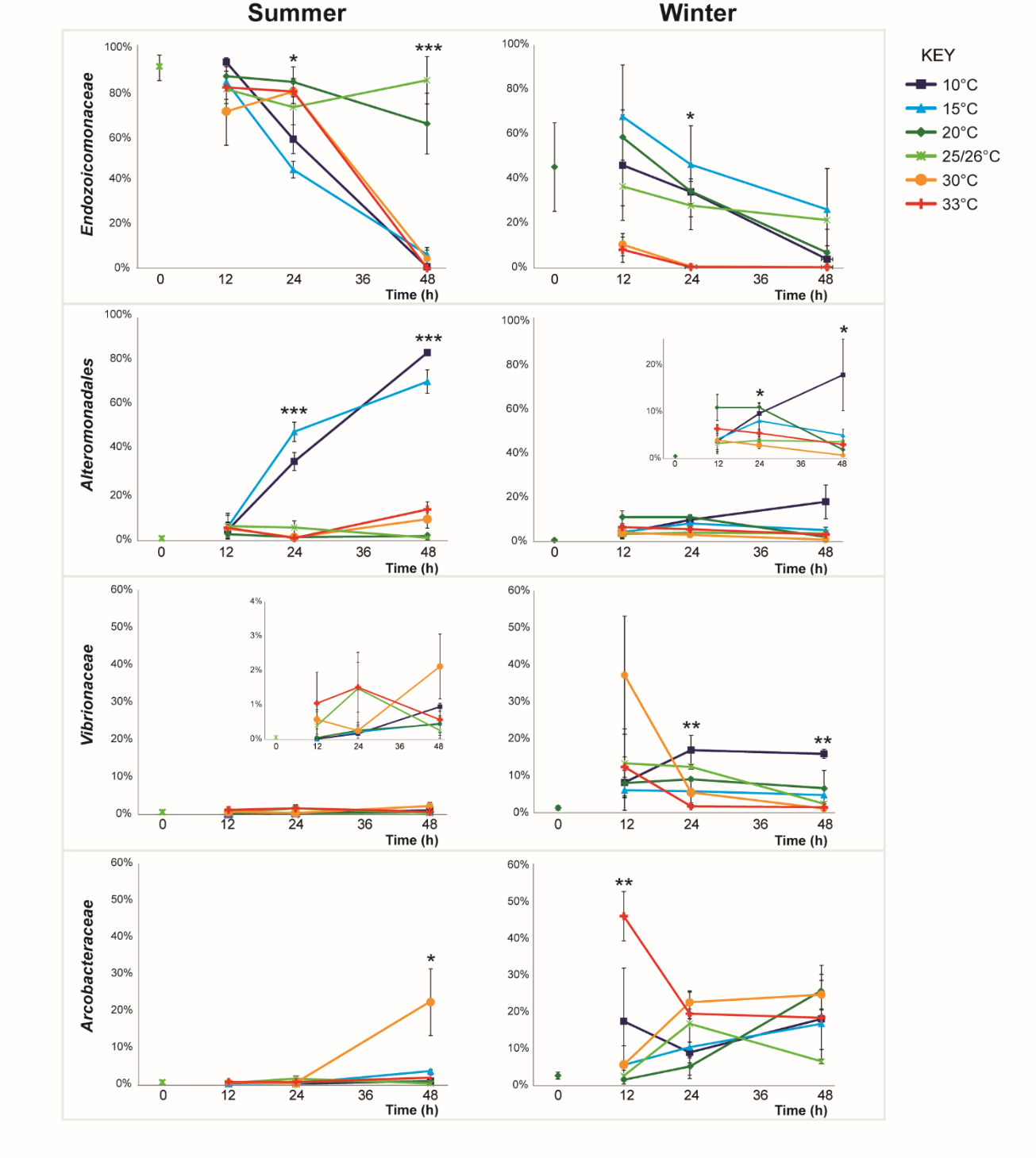


**Supplementary Figure S3.** Xyplot for relative abundance of 4 bacterial taxonomies over time, *Endozoicomonaceae*, *Alteromonadales*, *Vibrionaceae*, and *Arcobacteraceae*. Average and standard error of mean of relative abundances of 3 biological replicates are in the y axis, whereas the x axis represents time after treatment. Differences among temperature treatments at each sampling time: *p*-value < 0.05 (*); < 0.01 (**); < 0.001 (***).

**Figure S4**


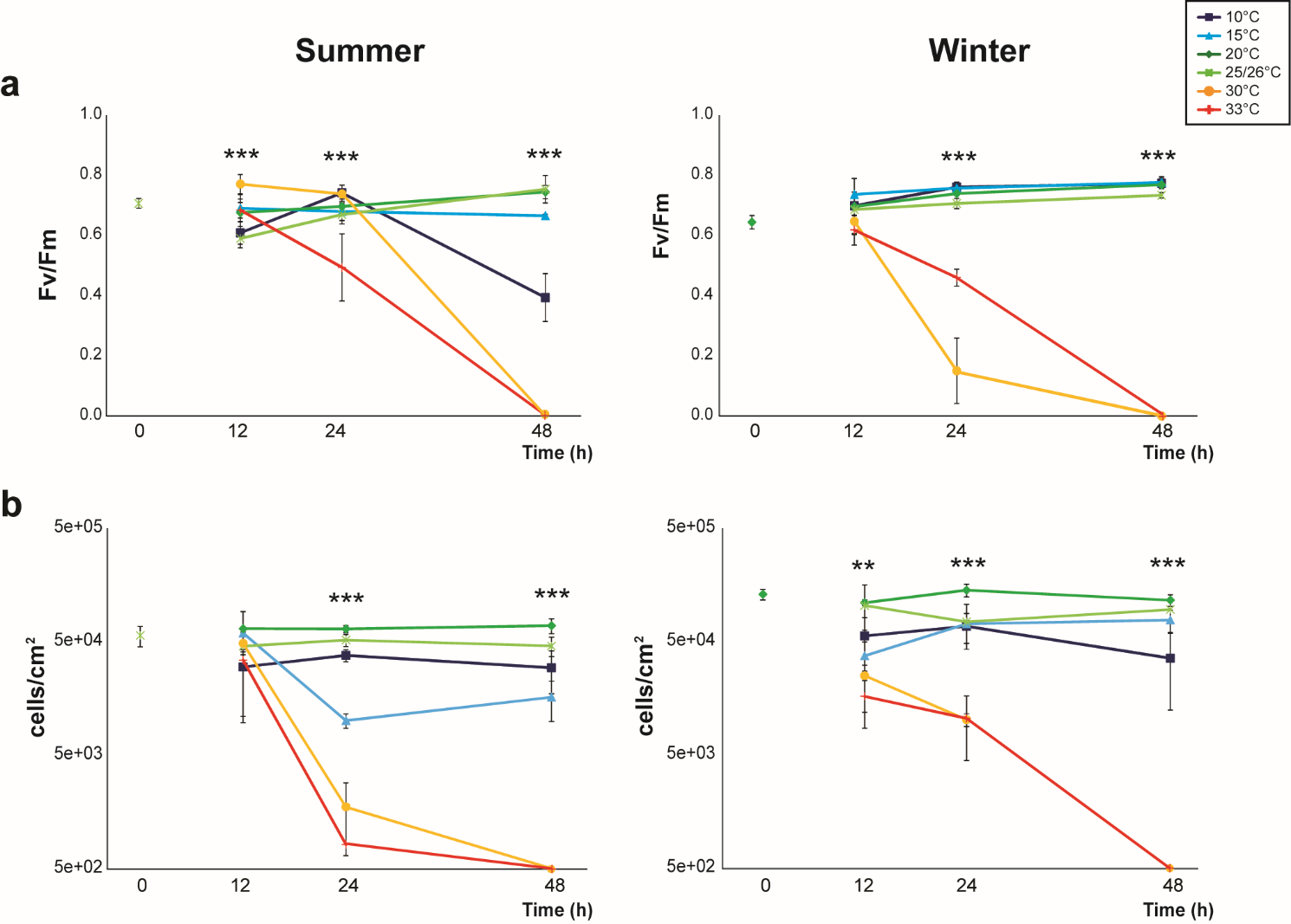


**Supplementary Figure S4.** Xyplot for (**a**) photosynthetic efficiency and (**b**) density of *Symbiodinium* over time. Average and standard deviation of 3 biological replicates are along the y axis, whereas the x axis represents time after treatment. Differences among temperature treatments at each sampling time: *p*-value < 0.05 (*); < 0.01 (**); < 0.001 (***).

Figure S5

**
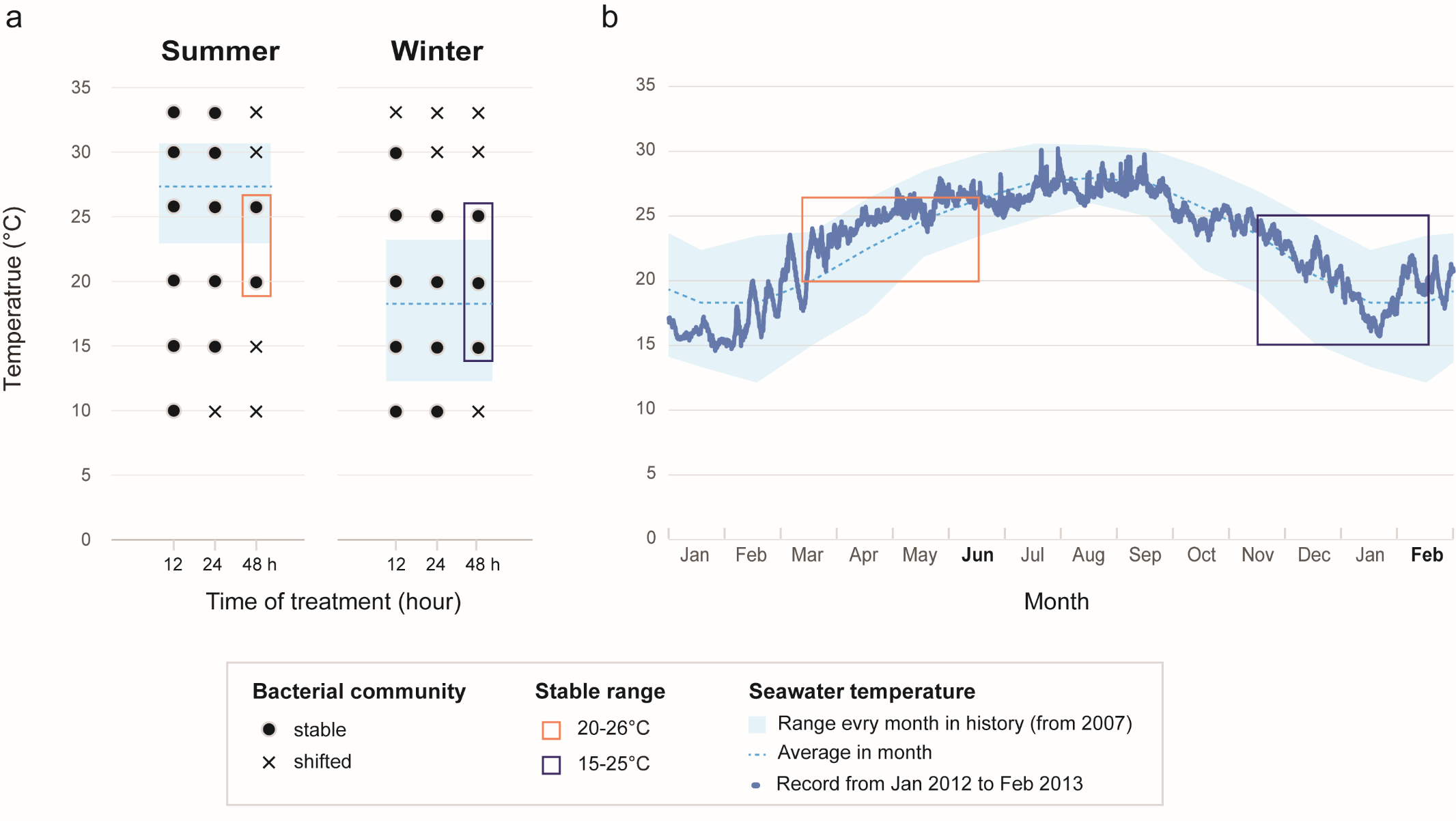
**

**Supplementary Figure S5 a.) Stable bacterial community under various temperature stresses at each sampling time.** Treatments with stable coral-associated bacterial communities are marked as filled circles, whereas shifted communities are marked as a cross. Orange squares represent the range of temperature with stable coral-associated bacteria community after 48 h of treatment in summer, and dark blue squares represent the temperature range with stable bacterial community in winter. A light blue background indicates historical seawater temperature range in summer (June to August) and winter (December to February). Dashed blue lines indicate average temperatures in summer and winter. **b.) Seawater temperature profile in Penghu.** Historical temperature ranges in each month from 2007 to 2014 (light blue background) ^68^, temperature average in each month from 2007 to 2014 (dashed blue line) ^68^, and record temperatures from January 2012 to February 2013 (blue line). Months in which samples were collected are bolded. Orange and dark blue squares indicating temperature ranges were consistent with seawater temperature history 3 mo before the experiment. Therefore, we inferred that the bacterial community was stable in seawater temperature treatments, because they had experienced and acclimated to temperature fluctuation within the 3 mo before samples were collected.
